# Theory‐based promotion of diet and transportation behavior change to reduce carbon footprint among students: Randomized parallel trial of the GROW app

**DOI:** 10.1111/aphw.70161

**Published:** 2026-06-12

**Authors:** Dario Baretta, Carole Lynn Rüttimann, Jennifer Inauen

**Affiliations:** ^1^ Institute of Psychology University of Bern Bern Switzerland

**Keywords:** behavior change, digital intervention, Health Action Process Approach, individual carbon footprint, mitigation behaviors

## Abstract

Adopting a low‐emission diet and choosing low‐emission transportation modes are among the most effective strategies for mitigating one's individual impact on climate. However, interventions targeting these behaviors often fall short because they focus primarily on motivation, neglecting volitional processes. Guided by the Health Action Process Approach, this double‐blind randomized parallel trial tested whether a digital intervention addressing both motivational and volitional determinants of behavior was more effective in reducing diet‐ and transportation‐related carbon footprints than an intervention addressing motivation only. The intervention was delivered via the GROW app, which included two versions: a motivational version (goal setting and feedback) and a motivational + volitional version (additional techniques such as action planning and problem solving). Participants (*N* = 226; 97% students) used either version of the app for 5 weeks, reporting daily animal‐based food consumption (e.g., red meat and poultry) and transportation behaviors (e.g., bike and car). Psychological determinants (e.g., action planning and action control) were measured weekly. Multilevel models showed an overall reduction in diet‐related carbon footprint (*B* = −0.1, 95% CI [−0.02, −0.01]); however, this decrease did not differ between intervention groups. No changes emerged for transportation‐related or total carbon footprints. Action planning and action control emerged as the strongest correlates of lower diet‐related carbon footprint. The findings show preliminary indication of the efficacy of digital behavior change interventions for reducing diet‐related carbon footprint. Structural measures addressing opportunities and barriers in the physical environment may be needed to reduce transportation‐related emissions.

## INTRODUCTION

Climate change is one of the most crucial issues that our global society is facing. It is mostly caused by uncontrolled emissions of greenhouse gas (GHGE; e.g., carbon dioxide [CO2]) produced by human activity (IPCC, 2023). Deep cuts in emissions require, among others, changes in human behavior and consumption patterns (IPCC, 2014). Those behaviors that individuals can modify to limit GHGE and reduce their carbon footprint are referred to as mitigation behaviors (Gifford, 2014). Evidence suggests that adopting a low‐emission diet—particularly by reducing the consumption of animal‐based foods—and choosing low‐emission modes of transportation (e.g., cycling or traveling by train instead of driving) are among the most impactful mitigation behaviors at the individual level (Bernard, 2019; Chevance et al., 2021; Godfray et al., 2018; IPCC, 2023; Willett et al., 2019). In certain forms, such as reduced meat consumption and active transportation, these two behaviors are also strongly linked to human health, as they help prevent non‐communicable diseases (Aleksandrowicz et al., 2016; Rohrmann et al., 2013; Whitmee et al., 2024). Therefore, interventions targeting low‐emission diets and transportation modes are a priority for curbing climate change and promoting human health (Inauen et al., 2021).

### The impact of diet and transportation on climate change and health

Among these mitigation behaviors, dietary choices have received increasing attention because food production—especially animal‐based foods—contributes substantially to global GHGE (von Braun et al., 2023; Xu et al., 2021). The climate impact of animal‐based foods is not uniform. Red meat from ruminants such as beef and lamb generates substantially higher emissions than red meat from nonruminant mammals such as pork, whereas white meat such as poultry typically has a lower footprint (Aleksandrowicz et al., 2016; Godfray et al., 2018; Willett et al., 2019). Fish and seafood can also be considered a type of meat (Boler & Woerner, 2017, but also see Ioannidou et al., 2024). Whereas fish is usually associated with emissions comparable to poultry, some seafood products (e.g., lobster) have substantially higher footprints, in some cases equivalent to or exceeding those of red meat. Other animal‐based food products, such as dairy, are generally associated with lower GHGE than red meat, although the impact varies by product type (Mertens et al., 2019).

In terms of direct health effects, in high‐income countries, red and processed meat intake is associated with colorectal cancer, whereas processed meat is further linked to stomach cancer, cardiovascular disease, and diabetes (Godfray et al., 2018). Despite these risks, findings from the Global Dietary Database show that in 2018 only 3.8% of countries worldwide met the Eat–Lancet target for red meat consumption consistent with healthy diets from sustainable food systems (≤98 g/week of combined unprocessed and processed red meat) (Miller et al., 2022; Willett et al., 2019). Globally, adults consume an average of 56 g of unprocessed red meat and 17 g of processed meat per day, whereas in high‐income regions such as Western Europe, the averages are 50 and 31 g/day, respectively. Even less stringent recommendations—such as the World Cancer Research Fund (WCRF) guideline to eat as little processed meat as possible and the United Kingdom's Scientific Advisory Committee on Nutrition (UK SACN) and WCRF limit of ≤70 g/day of red meat (Scientific Advisory Committee on Nutrition, 2011; World Cancer Research Fund, 2018)—are rarely met. For example, in Western European countries including the Netherlands, Ireland, and the United Kingdom, consumption of red and processed meat exceeds these recommendations (Cocking et al., 2020).

Transportation (i.e., travel from A to B for work, study, or daily activities) is another major source of emissions (IPCC, 2023). In 2019, the transport sector accounted for 15% of net global GHGE, with road transport alone responsible for about 10% of net global GHGE in 2020. Private light‐duty vehicles (e.g., cars) represented 53.2% of all trips in 2015, and their use must decrease by 4–14% below business‐as‐usual levels by 2030 (IPCC, 2023; United Nations Environment Programme, 2022). In contrast, active transportation modes such as walking and cycling have little to no climate impact while also playing a fundamental role in improving health outcomes (Ding et al., 2025; Mueller et al., 2015; Oja et al., 2011; Paluch et al., 2022).

### Promoting low‐emission diets and transportation modes

A wide range of interventions has been developed to promote various mitigation behaviors; however, their overall effectiveness remains limited (Nisa et al., 2019; Rau et al., 2022). When it comes to supporting the reduction of animal‐based food consumption, a recent systematic review concluded that although many studies have examined the drivers of meat consumption and the barriers to its reduction, few provide evidence for effective strategies to reduce meat consumption (Kwasny et al., 2022). Regarding transportation, a systematic review and meta‐analysis on car use showed no compelling evidence of effectiveness of behavioral interventions in decreasing the frequency of car use or increasing the proportion of journeys by more active travel modes (Arnott et al., 2014). One potential explanation is that interventions targeting these two mitigation behaviors predominantly rely on strategies such as providing information to increase knowledge or attempting to elicit emotional responses—approaches that have been shown to have limited impact (Kwasny et al., 2022; Nisa et al., 2019; Pawluk De‐Toledo et al., 2022). Although these intervention strategies may raise awareness and motivation to change behavior, evidence suggests that motivation alone is often insufficient to achieve behavior change, a problem commonly referred to as the intention–behavior gap (Sheeran, 2002; Sheeran & Webb, 2016).

To address this gap, behavior change theories highlight the importance of volitional determinants that influence whether intentions translate into action (e.g., Bandura, 1982; Gollwitzer, 1999; Schwarzer, 2008). The Health Action Process Approach (HAPA; Schwarzer, 2008) explicitly integrates both motivational and volitional processes. In the motivational stage, individuals form the intention to change their behavior when they perceive a health risk associated with their current behavior, have positive outcome expectancies related to changing their behavior, and are confident in their ability to master the target behavior (i.e., self‐efficacy; Bandura, 1982). In the volitional stage, these intentions are more likely to be enacted when supported by concrete action planning (when, where, and how), coping planning (the ability to make plans for overcoming obstacles), coping self‐efficacy (confidence in overcoming obstacles), and action control, which entails an individual's awareness of their goals, self‐monitoring of their behavior, and regulatory efforts (Sniehotta et al., 2005). The HAPA model has been shown to explain several health behaviors, including diet and physical activity (Zhang et al., 2019). Further, the HAPA model has shown efficacy in changing diet (Maghsoodlo et al., 2025; Zhou et al., 2013) and physical activity (Maghsoodlo et al., 2025; Silva‐Smith et al., 2024) in the context of health promotion. However, to the best of our knowledge, the HAPA framework has not been extended to high‐impact mitigation behaviors such as adopting low‐emission diets and transportation modes.

Because adopting a low‐emission diet and choosing low‐emission transportation modes are highly relevant mitigation behaviors, the question arises whether both should be tackled at the same time. Although there is little evidence on the effectiveness of a multiple behavior change strategy for mitigation behaviors, insights can be drawn from other health‐related interventions. In this field, the superiority of multiple behavior change interventions over single‐behavior ones is not yet clear, and little is known about which theoretical frameworks are best suited to guide them (James et al., 2016; Nigg & Long, 2012; Prochaska & Prochaska, 2011). Still, some studies suggest that targeting behaviors such as physical activity and diet at the same time can be more effective than usual care (Hyman et al., 2007; King et al., 2013; Vandelanotte et al., 2007), in particular when structured around a common health‐related goal, such as cancer prevention (Nigg & Long, 2012). These insights suggest that there may be value in leveraging multiple behavior change approaches to simultaneously target mitigation behaviors with the shared goal of reducing GHGE, such as dietary and transportation choices. Nonetheless, since changing several behaviors at once can be burdensome, it is important to support individuals' engagement and sense of control (Perski et al., 2018), for example, by allowing participants to decide how many mitigation behaviors they wish to address at a given time.

Beyond the content of interventions promoting low‐emission diets and transportation modes, there is the question of how such interventions should be delivered. One opportunity lies in the use of digital interventions, as this mode of delivery combines scalability with personalized support (Kavanagh, 2020), offering a cost‐effective way to promote sustainable behaviors to a broad audience. Recent research highlights the growing potential of such digital tools in encouraging pro‐environmental actions (e.g., Boncu et al., 2022; Morganti et al., 2017). However, past studies of digital interventions to change mitigation behaviors largely lack a theoretical basis and often fail to adequately explain how or why they would change behavior (Mosca et al., 2024).

### Aim of the study

The main focus of this study was to test whether the *additional contribution of volitional components* into a mobile app‐based intervention (the *GROW app*) designed to promote low‐emission dieting by reducing the consumption of animal‐based foods and low‐emission transportation as mitigation behaviors leads to greater reduction in individuals' carbon footprints compared to providing motivational components alone. Overcoming the limitations of previous studies, which often had a motivational focus or lacked a solid theoretical foundation (see systematic reviews by Arnott et al., 2014; Kwasny et al., 2022; Mosca et al., 2024; Nisa et al., 2019), our intervention was based on the HAPA model and particularly built on the idea that volitional determinants of behavior play a crucial role in behavior change. We hypothesized that targeting both the motivational and volitional phases of behavior change (motivational + volitional group) would be more effective in promoting mitigation behaviors compared to a motivational‐only intervention (motivational group).

In both groups, we expected a significant decrease over time in diet‐related carbon footprint (H1a), transportation‐related carbon footprint (H1b), and their sum (i.e., total carbon footprint; H1c) during the usage of the GROW app. Additionally, we expected group differences in decreases in carbon footprint, hypothesizing greater decreases in the motivational + volitional group in diet‐related (H2a), transportation‐related (H2b), and total carbon footprint (H2c) compared to the motivational group. Finally, based on theoretical propositions from the HAPA model, we explored whether the intervention effects on carbon footprint were mediated by self‐efficacy (H3a), action planning (H3b), coping planning (H3c), and action control (H3d), which are the main psychological determinants characterizing the volitional phase.

## METHODS

**FIGURE 1:**
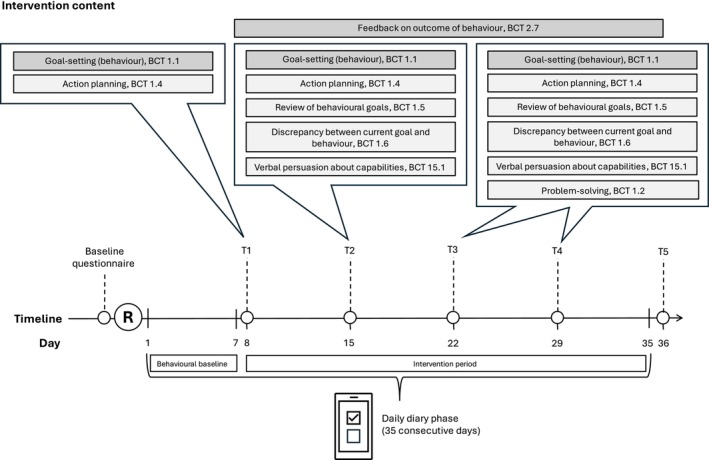
Trial timeline. Abbreviation: R, randomization. *T*0 represents the timepoint of the baseline questionnaire. *T*1–*T*4 represent questionnaires during intervention delivery, whereas *T*5 was the post‐intervention questionnaire. The psychological determinants were assessed during *T*1–*T*5. Behavior change techniques (BCTs) are named following the behavior change technique taxonomy (Michie et al., 2013). BCTs illustrated in light gray were presented to both intervention groups, whereas BCTs in dark gray were presented to the motivational + volitional intervention group only.

The study design was a double‐blind randomized parallel trial that included two experimental groups: a motivational group and a motivational + volitional group. Participants were randomly assigned to either group with equal probability (1:1 allocation ratio). The total study duration for each participant was 37 days, including a 35‐day daily diary phase during which diet‐ and transportation‐related carbon footprints were assessed (Figure 1). After a 1‐week baseline period, intervention content was delivered at the end of each week of the 4‐week intervention phase. The reporting of the trial is in line with the CONSORT (Consolidated Standards of Reporting Trials) 2025 standards (Hopewell et al., 2025; see Table S1) and follows the TIDieR (Template for Intervention Description and Replication) guidelines (Hoffmann et al., 2014, see Table S2). The ethics commission of the Faculty of Human Sciences at the University of Bern, Switzerland, approved this study. The study protocol was preregistered on April 3, 2024 (10.17605/OSF.IO/BF594). The study was conducted in Switzerland (Box S1 provides information on the travel‐related infrastructures and figures in this country).

### Participants

Participants were eligible if they were at least 18 years old and had sufficient German language skills. Exclusion criteria were a current or past diagnosis of eating disorders and following a vegan or vegetarian diet. Persons who did not provide informed consent were excluded.

The required sample size was 176 participants. Given the multiple assessment of 35 days, the total observations (176 participants × 35 daily carbon footprint measurements) were estimated to be enough to detect a medium standardized effect size (product–moment correlation [*γ*_*std*_] = .30) for both the Level 1 direct effects (H1a–H1c) and cross‐level interactions (H2a–H2c) in a multilevel model. This a priori power analysis was calculated using the R *simr* package (Green & MacLeod, 2023) with the code adapted from Arend and Schäfer (2019). To account for an anticipated 20% attrition rate during the study, the final target sample size was increased to 220 participants.

### Intervention groups

Participants allocated to the motivational group were exposed to the following Behavior Change Techniques (BCTs; Michie et al., 2013): 1.1 Goal Setting and 2.7 Feedback on Outcomes of Behavior. Participants in the motivational + volitional group received the same BCTs as the motivational group, in addition to the following techniques: 1.2 Problem Solving, 1.4 Action Planning, 1.5 Review of Behavioral Goals, 1.6 Discrepancy Between Current Behavior and Goal, 15.1 Verbal Persuasion About Capability, and 3.3 Social Support (Emotional). These additional BCTs were selected based on their theorized effect on key volitional‐phase behavioral determinants, including coping self‐efficacy, action planning, coping planning, and action control (Rhodes et al., 2020; Warner & French, 2020; Webb & de Bruin, 2020) (see Table S3). The BCTs were translated into features of the GROW app by triangulating theory‐specific guidelines (e.g., Epton & Armitage, 2020; Rhodes et al., 2020), materials from previous behavior change interventions implementing the same BCTs (e.g., Baretta et al., 2025; Lee et al., 2017), and a prior review examining the application of these BCTs in commercial behavior change smartphone apps (Baretta et al., 2019). For a detailed description of how these BCTs were presented to the participants, see Tables S4 and S5. For a timeline indicating in which intervention module (*T*1–*T*4) each BCT was introduced during the trial, see Figure 1. Before launching the study, both versions of the GROW app were extensively tested by the study team to identify and resolve technical issues. The app was also piloted with users to ensure clarity of instructions and to verify that the content was delivered and perceived as intended.

The intervention content was delivered via participants' personal smartphones through the GROW app, which was specifically designed for this study using the Self‐Help app development platform of the Faculty of Human Sciences of the University of Bern. The intervention was delivered from *T*1–*T*4. At the end of each week, participants were asked to complete an intervention module that included the BCTs according to their assigned experimental group, targeting both dietary and transportation behaviors. Engagement with the dietary modules was mandatory, whereas the transportation modules were optional, depending on whether participants set transportation‐related goals during the respective intervention week. This optional engagement deviates from the preregistered protocol but was deliberately implemented before the start of the trial to reduce participant burden and minimize potential dropout. For the same reasons, we chose to address dietary and transportation behaviors simultaneously rather than sequentially. A sequential approach would have extended the intervention timeline and been less relevant for participants who already relied extensively on active transportation. Consequently, engagement with the transportation‐related module was lower compared to the diet‐related one, requiring adjustment to the data analysis plan (see Section 2.5.2).

Finally, to enhance participant engagement with the app and introduce novelty throughout the 4‐week intervention period, all participants received entertaining daily content, including tips, short readings, and quizzes related to environmental and health aspects of diet and transportation (see Table S6).

### Measures

Self‐reported age, sex, and sociodemographic characteristics (such as living situation and income) were assessed in the baseline questionnaire. Additionally, eating routines were assessed by asking participants how often they (i) prepare their meals themselves and (ii) eat outside. Responses were recorded on a Likert scale ranging from 0 (*never*) to 4 (*always*). Access to transportation options was assessed by asking participants about their available transportation options (e.g., owning a car and having a train pass) and the distance to the nearest public transport station or stop.

The primary outcomes, diet‐ and transportation‐related carbon footprint, were calculated daily during the baseline week and the subsequent four intervention weeks, based on participants' self‐reported dietary and transportation behavior. The secondary outcomes, which include the target volitional psychological determinants of the intervention effect on carbon footprint based on the HAPA model, were assessed weekly.

#### Primary outcomes: Carbon footprint

##### Diet‐related carbon footprint

Daily diet‐related carbon footprint was expressed in kilogram of GHGE per kilogram of food and was derived from participants' self‐reported consumption of animal‐based products across eight food categories (for specific self‐report items see Table S7 and Figure S1). For each category, participants reported the type and amount of products consumed, expressed in number of servings. Serving sizes were defined according to the recommendations of the Swiss Society for Nutrition (SGE‐SSN, 2024). The self‐reported food items corresponded to eight categories derived from the diet‐related carbon footprint analysis: (1) ruminant meat (e.g., beef), (2) pork and processed meat, (3) white meat (e.g., chicken), (4) fish, (5) seafood, (6) eggs, (7) cheese, and (8) other dairy products (e.g., yogurt and milk). Contrary to what was stated in the registered protocol, we ultimately decided to consider fish and seafood as two distinct food categories, as they are associated with substantially different carbon footprints. To estimate diet‐related carbon footprint, kilogram of GHGE per kilogram of animal‐based food products was sourced from the publicly available SHARP Indicators Database (accessed July 22, 2022; Mertens et al., 2019). A three‐step classification process was used: (1) Items were ranked by GHGE; (2) grouped according to typical consumption patterns based on the Swiss Society for Nutrition (SGE‐SSN, 2022) guidelines; and (3) average GHGE per kilogram was calculated within each category. For categories such as fish and seafood, eggs, cheese, and other dairy products, upper‐end outliers were winsorized at the 90th percentile to ensure representative averages. GHGE per serving was calculated using SGE‐SSN‐recommended serving sizes, and participants' daily diet‐related carbon footprints were estimated by multiplying reported servings by the respective GHGE/kg values (for details, see Box S2 and Table S8).

##### Transportation‐related carbon footprint

Daily transportation‐related carbon footprint was estimated based on participants' self‐reported transportation behavior, collected through 14 items assessing the transportation mode(s) (e.g., by foot and bicycle), and distance traveled (in kilometers) (for specific self‐report items, see Table S9 and Figure S2). Transportation was defined as travel from one location to another (e.g., to work, the supermarket, or a holiday destination), excluding exercise or leisure activities such as jogging or recreational cycling. To estimate transportation‐related carbon footprint, GHGE per passenger kilometer (GHGE/pkm) for each transport mode was sourced from the mobitool database (file “mobitool‐faktoren‐v2.1‐short‐v2,” accessed July 12, 2022), maintained by Switzerland's Federal Office for the Environment. For ease of reporting, transport types were grouped into six categories: (1) foot, (2) bike (normal bike and e‐bike), (3) motorbike (scooter, e‐scooter, and average motorbike), (4) car (diesel, petrol, hybrid, electric, or weighted average if unspecified), (5) public transportation, and (6) airplane (various classes and distances). Daily transportation‐related carbon footprint was calculated by multiplying the reported distance for each transport type by its corresponding GHGE/pkm (see Table S10 for details).

##### Total carbon footprint

Total carbon footprint was calculated as the daily sum of diet‐ and transportation‐related carbon footprints.

#### Secondary outcomes: Social‐cognitive behavioral determinants

The target volitional determinants (i.e., action planning, coping planning, action control, and self‐efficacy) and behavioral intention were assessed using self‐report questionnaires, with one set of items each addressing dietary and transportation behavior. These questionnaires were adapted from existing literature to align with the target mitigation behaviors. Participants were thoroughly informed about the specific definitions of environmentally sustainable eating and transportation. The items for each scale were averaged.

##### Action planning

Action planning as captured through six items (three per behavior) adapted from Allan et al. (2013). Responses were given on a 5‐point Likert scale ranging from 0 (*not at all true*) to 4 (*exactly true*; McDonald's *ω* = 0.82 for diet‐related items at *T*1 and McDonald's *ω* = 0.90 for transportation‐related items at *T*1). An example item reads: “I already planned concretely how I am going to eat pro‐environmentally.”

##### Coping planning

To assess coping planning, participants responded to six items (three per behavior; e.g., “I already planned concretely what to do in difficult situations in order to stick to my goal to eat pro‐environmentally”), drawing on adaptations from Reyes Fernández et al. (2016) and Schwarzer (2008). The same 5‐point scale was used (McDonald's *ω* = 0.81 for diet‐related items at *T*1 and McDonald's *ω* = 0.84 for transportation‐related items at *T*1).

##### Action control

This determinant was measured using 12 items (six per behavior) adapted from Sniehotta et al. (2005). Items evaluated participants' ongoing self‐regulatory efforts over the past week, such as follows: “During the last week, I constantly monitored myself whether I was eating pro‐environmentally to reach my dietary goal.” Again, responses were made on the 0–4 Likert scale (McDonald's *ω* = 0.83 for diet‐related items at *T*1 and McDonald's *ω* = 0.85 for transportation‐related items at *T*1).

##### Coping self‐efficacy

Finally, self‐efficacy was examined using 20 items (10 per behavior), based on work by Schwarzer (2008) and Schwarzer et al. (2007). Items assessed confidence in maintaining pro‐environmental behaviors despite various barriers. For example: “I can stick to eating pro‐environmentally even if I am stressed out.” All items used the same 5‐point response format (McDonald's *ω* = 0.90 for diet‐related items at *T*1 and McDonald's *ω* = 0.93 for transportation‐related items at *T*1).

##### Intention

Intention was measured on a 5‐point Likert scale with items adapted to diet and transportation (two items per behavior) from Reyes Fernández et al. (2016), ranging from 0 (*not at all*) to 4 (*very strongly*). An example item reads: “To what extent do you intend to eat pro‐environmentally during the upcoming week?”

### Procedure

Participants were recruited from the University of Bern's participant pool, a university mailing list, and the department website. Interested individuals signed up either through the participant pool or by email and were redirected to the study page on Qualtrics. There, they received detailed study information and completed an eligibility and consent survey, including an electronic consent form. After providing informed consent, participants were emailed a registration code and instructed to download the GROW app from the Google Play Store or App Store. Once registered in the app, they could select their preferred starting date for the study, which marked the completion of the baseline questionnaire.

Following the baseline questionnaire, participants were randomized via a simple randomization into one of two experimental groups. Randomization was implemented through the Qualtrics software, ensuring allocation concealment. Although researchers responsible for monitoring and data analysis had access to group allocation after assignment, this information was only consulted in cases of technical issues reported by participants. This limited access did not compromise the integrity of the double‐blind design, as the intervention was automatically delivered via the app. On the day after randomization, participants began the first daily diary entry, which assessed diet‐ and transportation‐related carbon footprint. Participants were instructed to use the GROW app to report their daily consumption of animal‐based products and transportation behavior each evening before going to bed for the following 35 days. The app also sent a daily notification to participants as a reminder to complete their daily reports. The first 7 days served as a behavioral baseline, after which the intervention phase commenced. Psychological determinants were assessed weekly from *T*1 to *T*5 (see Figure 1). Additional notifications were sent on specific study days to prompt participants about the weekly psychological questionnaires and intervention sessions. All questionnaires were developed in Qualtrics and embedded into the GROW app via an application programming interface. Participants' data were securely stored on Qualtrics servers. As an incentive, three gift vouchers (each worth 100 CHF for a zero‐waste store) were raffled among participants who completed the study.

### Data Analysis

Analyses were conducted with the R software, Version 4.4.2 (R Core Team, 2024). R code and data are available online (at https://osf.io/gakxm).

#### Hypotheses Testing

Data were analyzed using multilevel modeling (MLM) with the R package *lme4* (Version 1.1.35.5; Bates et al., 2015), accounting for the nested structure of repeated measurements within participants. A significance threshold of *p* < .05 was applied to all statistical tests. Influential cases (i.e., outliers) were identified using Cook's distance, and those exceeding the threshold of 1 were removed (Fox, 1991).

##### Primary analysis

To test H1a–c and H2a–c for diet‐related, transportation‐related, and total carbon footprint (dependent variables), MLMs (one for each dependent variable) included fixed effects for time, experimental group, and their interaction (group × time). For the time variable, Days 1–7 were coded as 0, representing the behavioral baseline, whereas Days 8–35 were coded sequentially from 1 to 28, corresponding to the progressive days of the intervention phase. Random intercepts were specified for each participant to account for individual baseline differences. We also tested models with a random slope for time but encountered convergence issues, and the models' performance, as assessed by the Bayesian Information Criterion (BIC), did not show improvement compared to random intercept‐only models. H1a–c were considered supported if the fixed effect of time was negative and statistically significant, indicating a decrease in carbon footprint over time. H2a–c were considered supported if the interaction between time, and intervention group was statistically significant and indicated a greater reduction in carbon footprint in the motivational + volitional group compared to the motivational group.

##### Mediation analysis

In Hypotheses H3a–d, we preregistered mediation analyses to examine whether potential intervention effects on carbon footprint would be mediated by changes in psychological determinants: self‐efficacy (H3a), action planning (H3b), coping planning (H3c), and action control (H3d). These analyses were specified a priori to be conducted only in the presence of a significant intervention effect on the outcome. Under this condition, the analytical approach was registered as a 2‐1‐1 longitudinal mediation analysis (Berli et al., 2021), using MLM to account for the nested data structure of repeated measurements within participants. Additionally, because psychological determinants were assessed weekly (at five time points) and carbon footprint was measured daily (across 35 time points), we computed the mean weekly carbon footprint score to align both variables at the same temporal resolution. To test the mediation hypothesis, we first checked the prerequisite of an intervention effect (group × time) on each psychological determinant (Path *a* in the mediation analysis) (MacKinnon et al., 2007) and whether each determinant was associated with carbon footprint (Path *b*). In examining Path *b*, social‐cognitive determinants were between‐ and within‐person centered. Indirect effects were calculated only in case both Paths *a* and *b* were significant.

#### Divergence from study protocol

Participation in the weekly transportation‐related intervention module was optional, leading to lower engagement rates compared to the diet‐related intervention module. Additionally, engagement with the transportation module was higher in the motivational group (see Section 3.2). To mitigate the risk of bias arising from this differential engagement, we adjusted for it when testing our hypotheses. Specifically, we included engagement with the transportation‐related intervention module as a covariate when testing Hypotheses H1b and H1c (time effect) and H2b and H2c (group × time effect) concerning total‐ and transportation‐related carbon footprints, as well as H3a–d concerning transportation‐related social‐cognitive determinants. This was done by adding a dummy variable to the model that specified whether the daily assessment followed a completed weekly transportation‐related intervention module (dummy value = 0) or not (dummy value = 1).

#### Sensitivity analysis

For sensitivity analyses, primary models for H1 and H2 (effects on carbon footprint) were estimated again using imputed datasets (Tables S11 and S12). Missing values in daily carbon footprint measures (diet‐ and transportation‐related) were imputed using the Kalman Filter method via the *imputeTS* package (Version 3.3; Moritz & Bartz‐Beielstein, 2017), which is well suited for univariate time series data (Gómez & Maravall, 1994). We imputed missing values only under conditions where there were no more than three consecutive missing values in a row. Attempts to impute longer gaps of missing values would result in unrealistic decreases in the imputed value variance. In contrast, for H3 (mediation analysis), missing values in social‐cognitive determinants were handled using the Last Observation Carried Forward (LOCF) approach, as the Kalman Filter method is not appropriate for non‐intensive longitudinal data. We chose LOCF, as it offers a parsimonious yet effective strategy for preserving statistical power and Type I error control (Overall et al., 2009).

## RESULTS

**FIGURE 2:**
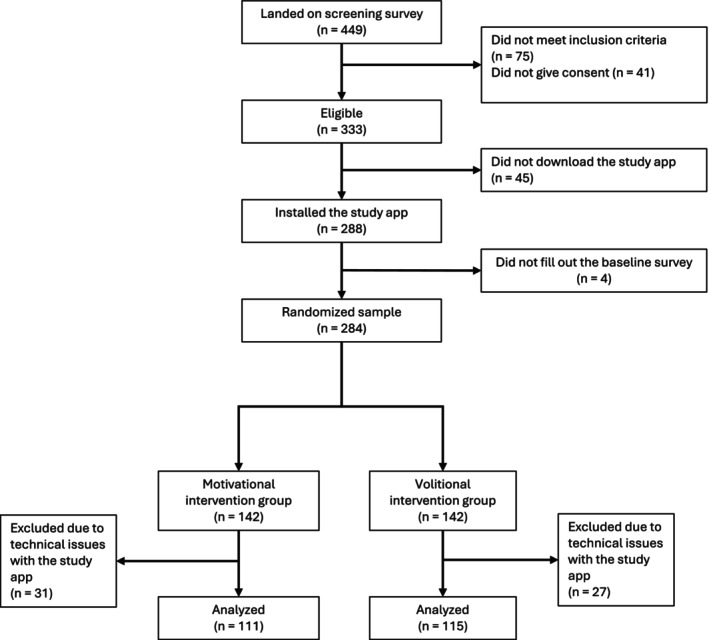
Participant recruitment flow.

The recruitment for the GROW trial began on April 14, 2024, and ended on July 7, 2024. Post‐intervention assessments occurred between May 21, 2024, and August 12, 2024. Overall, 284 participants were recruited and randomized to one of the two intervention groups. Fifty‐eight participants (20%) were excluded from the analysis due to technical issues in data collection (e.g., personal goals not displayed correctly in the app; for more information about goal setting, see Tables S4 and S5). To make up for the technical dropouts, we continued recruiting until a final analytical sample of 226 participants was reached. The trial concluded when the last recruited participant completed the final assessment T5. Figure 2 shows the participants' flow through the trial.

### Descriptive statistics

Descriptive statistics for the analytical sample are presented in Table 1. Two hundred twenty (97%) were students from the University of Bern. The motivational and the motivational + volitional group did not significantly differ in sociodemographic characteristics, carbon footprint, or social‐cognitive determinants at baseline (see Tables 1 and S11).

**TABLE 1:** Sociodemographic characteristics, behavioral intention, and carbon footprint at baseline.

	Overall *N* = 226	Motivational group = 111	Motivational + Volitional group *N* = 115	Group differences (*p* value)^a^
Age				.329
Median (Q1, Q3)	22 (20, 23)	22 (21, 24)	22 (20, 23)	
Min, max	18, 59	18, 59	19, 35	
Sex^b^				.343
Male	38 (17%)	16 (14%)	22 (19%)	
Female	188 (83%)	95 (86%)	93 (81%)	
Living situation^b^				.810
Alone	20 (8.8%)	9 (8.1%)	11 (9.6%)	
With parents	116 (51%)	55 (50%)	61 (53%)	
With children/partner	23 (10%)	11 (9.9%)	12 (10%)	
Shared flat	66 (29%)	36 (32%)	30 (26%)	
Other	1 (0.4%)	0 (0%)	1 (0.9%)	
Monthly household Netto income (Swiss Francs)^b^				.353
Less than 2000	37 (20%)	17 (18%)	20 (21%)	
2001–4000	38 (20%)	23 (25%)	15 (16%)	
4001–6000	26 (14%)	15 (16%)	11 (12%)	
6001–8000	26 (14%)	14 (15%)	12 (13%)	
8001–10,000	24 (13%)	10 (11%)	14 (15%)	
More than 10,000	37 (20%)	14 (15%)	23 (24%)	
Missing	38	18	20	
Access to mobility options^b^				
Own a car	119 (53%)	57 (51%)	62 (54%)	.700
Own a motorbike	8 (3.5%)	4 (3.6%)	4 (3.5%)	>.999
Own a bike	150 (66%)	69 (62%)	81 (70%)	.188
Have an annual public transport pass^c^	153 (68%)	70 (63%)	83 (72%)	.143
Have a half fare card^d^	76 (34%)	40 (36%)	36 (31%)	.452
Intention				
Low‐emission diet				.407
Median (Q1, Q3)	3.00 (2.50, 3.00)	3.00 (2.00, 3.00)	3.00 (2.50, 3.00)	
Missing	1	0	1	
Low‐emission transportation				.278
Median (Q1, Q3)	3.00 (2.50, 3.50)	3.00 (2.50, 3.00)	3.00 (3.00, 3.50)	
Missing	1	0	1	
Carbon footprint^e^				
Diet‐related CF				.222
Median (Q1, Q3)	2.14 (1.47, 3.09)	2.38 (1.46, 3.38)	2.05 (1.47, 2.92)	
Min, max	0.34, 6.28	0.49, 6.20	0.34, 6.28	
Missing	12	8	4	
Transportation‐related CF				.958
Median (Q1, Q3)	1.6 (0.6, 3.5)	1.7 (0.5, 3.6)	1.6 (0.7, 3.5)	
Min, max	0.0, 260.1	0.0, 37.5	0.0, 260.1	
Missing	13	8	5	
Total carbon footprint (GHGE/kg)				.573
Median (Q1, Q3)	4.0 (2.7, 6.4)	4.3 (3.0, 6.4)	3.9 (2.5, 6.7)	
Min, max	0.5, 261.5	0.7, 41.9	0.5, 261.5	
Missing	12	8	4	

^a^ Wilcoxon rank sum test used for group differences in continuous variables, whereas Fisher's exact test used for group differences in categorical variables.

^b^ *n* (%).

^c^ It provides unlimited travel on most public transport in Switzerland.

^d^ It offers a 50% discount on most public transport in Switzerland.

^e^ Aggregated summary statistics from the first week of daily carbon footprint assessment, before participants attended any intervention session.

Abbreviation: CF, carbon footprint.

### Intervention fidelity

As an indicator of intervention fidelity, participants' engagement with the weekly intervention modules is displayed in Table S12. Weekly engagement with the diet‐/transportation‐related module was indicated by whether participants set a diet‐/transportation‐related carbon footprint goal for the upcoming week. For the mandatory diet‐related modules, the engagement rate ranged from 69% to 80% in the motivational group and from 66% to 75% in the motivational + volitional group over the course of the intervention. A Poisson regression analysis revealed no significant group differences (*B* = −0.04. *SE* = 0.08, *p* = .597). For the optional transportation‐related module, only a minority of participants set transportation‐related goals and thus engaged with the respective module, with percentages ranging from 21% to 32% in the motivational group and from 9% to 25% in the motivational + volitional group. Poisson regression revealed a significant group difference (*B* = −0.58. *SE* = 0.15, *p* < .001), with less engagement in the motivational + volitional group.

### Time and intervention effects on carbon footprint

**FIGURE 3:**
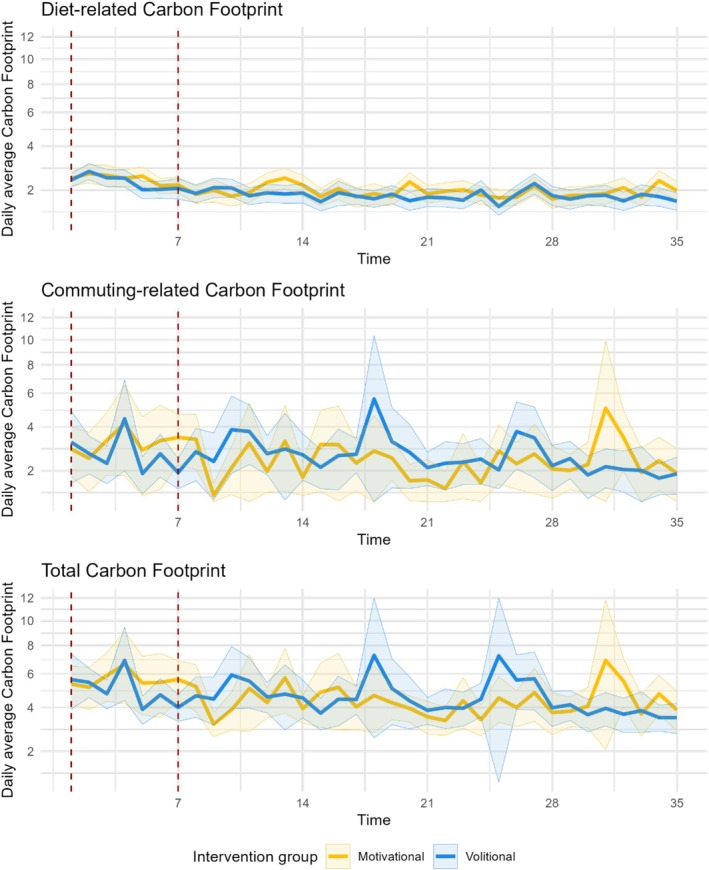
Change in carbon footprint over time. *Note*: The *y* axis is presented on a square root scale to better visualize the data distribution. Vertical red lines indicate the baseline phase, prior to intervention delivery.

Figure 3 shows how carbon footprint changed over the course of the study. As shown in Table 2, the main effect of time was statistically significant for the diet‐related carbon footprint outcome (H1a; *B* = −0.1, 95% CI [−0.02, −0.01]). Conversely, we did not find any time effect on transportation‐related carbon footprint (H1b) or the combined diet‐ and transportation‐related carbon footprint (H1c). The interaction between intervention group and time was not significant for diet‐related carbon footprint (H2a), transportation‐related carbon footprint (H2c), or total carbon footprint (H1c). Additionally, the frequency of engagement with the transportation‐related intervention module was not associated with transportation‐related and total carbon footprint. For the sensitivity analysis, the same analysis was conducted with missing value imputation. The results confirmed the same findings (H1b; *B* = −0.1, 95% CI [−0.02, −0.01]) (see Table S13). Further, an additional sensitivity analysis including a time‐varying covariate accounting for weekends and public holidays yielded consistent results. Transportation‐related and total carbon footprints were positively associated with weekends and public holidays (Table S14), suggesting that participants engaged in more carbon‐intensive transportation activities during these periods whereas the overall intervention effects remained unchanged.

**TABLE 2:** Time, group, and time‐by‐group effects on individual carbon footprint.

	Diet‐related carbon footprint	Transportation‐related carbon footprint	Total carbon footprint
Fixed effects			
(Intercept)	**2.24 (0.11)**	**2.77 (0.40)**	**4.77 (0.43)**
	**[2.02, 2.45]**	**[1.98, 3.55]**	**[3.93, 5.61]**
Time	**−0.01 (0.00)**	−0.02 (0.02)	−0.02 (0.02)
	**[−0.02, −0.01]**	[−0.06, 0.02]	[−0.06, 0.02]
Intervention	−0.11 (0.15)	0.18 (0.49)	0.08 (0.53)
	[−0.41, 0.19]	[−0.78, 1.14]	[−0.96, 1.11]
Time × intervention	−0.01 (0.00)	0.00 (0.02)	0.00 (0.02)
	[−0.01, 0.00]	[−0.05, 0.05]	[−0.05, 0.05]
Engagement with transportation module	–	0.09 (0.36)	0.52 (0.36)
	–	[−0.61, 0.79]	[−0.19, 1.23]
Random effects			
SD (intercept)	1.02	2.45	2.81
SD (residuals)	1.56	8.71	8.87
Model information			
Number of observations	6046	6035	6035
*R* ^2^ Marg.	0.009	0.000	0.002
*R* ^2^ Cond.	0.307	0.074	0.093
ICC	0.3	0.1	0.1

*Note*: Results of three multilevel models of diet‐related, transportation‐related, and total carbon footprint. Values presented in bold are significant based on their 95% confidence intervals. Number of observations differ across models due to the exclusion of outliers.

Abbreviations: ICC, intra‐class correlation; *R* ^2^ Cond., the part of the variance in the outcome explained by the full model; *R* ^2^ Marg., the part of the variance in the outcome explained by the fixed effects.

### Mediation of the intervention effect on carbon footprint by social‐cognitive determinants

The interaction between the intervention group and time was not significant for any diet‐ and transportation‐related target social‐cognitive determinants (Path *a* of the mediation analysis, Table S15). These results were also confirmed in the sensitivity analysis with missing value imputation (Table S16). Thus, the prerequisite for mediation was not met. We therefore refrain from testing mediation hypotheses and limit the following results to testing changes in psychological determinants over time and their relation to intervention outcomes. Target determinants increased over the course of the study, apart from transportation‐related self‐efficacy (Figures S3 and S4). Lastly, results indicated that engagement with the transportation‐related intervention modules was associated with an increase in all the psychological determinants.

Even though mediation was not supported, we provide results from the Path *b* analysis, which examines the association between social‐cognitive determinants and carbon footprint (Table S17). At the between‐person level, participants with higher action planning showed a larger reduction in diet‐related carbon footprint (*B* = −0.89, 95% CI [−1.73, −0.04]). At the within‐person level, increases in action control were associated with a reduction in diet‐related carbon footprint (*B* = −0.26, 95% CI [−0.45, −0.05]), whereas increases in coping planning were associated with an increase in diet‐related carbon footprint (*B* = 0.27, 95% CI [0.00, 0.55]). No significant associations were found for transportation‐related carbon footprint.

## DISCUSSION

In this parallel‐group randomized trial, we tested whether an intervention targeting motivational and volitional factors of the HAPA model leads to a reduction in individual carbon footprints related to diet and transportation behaviors compared to a motivation‐only intervention. Contrary to our Hypotheses H2a–c, we found no group differences. However, the results indicated that both the motivational and motivational + volitional groups had a small significant reduction in diet‐related carbon footprint over time, supporting Hypothesis H1a, with an average decrease of approximately 10 g of CO_2_‐equivalent per day per person, totaling 280 g by the end of the study. This represents a 13% reduction compared to the baseline median of diet‐related carbon footprint. No decrease was observed over time for transportation‐related or total carbon footprint (H1b and H1c). Mediation of intervention effects by social‐cognitive determinants could not be examined because no intervention effect was observed. Diet‐related carbon footprint was more strongly reduced among individuals with higher action planning and during weeks characterized by higher action control. No significant relationship was found between social‐cognitive determinants and transportation‐related carbon footprint.

One possible reason for the absence of a significant difference between the motivational and the motivational + volitional group is that baseline carbon footprints were relatively low, creating a floor effect with limited scope for further reduction. As noted by Rau et al. (2022), participants in pro‐environmental interventions often show limited additional change because they have already adjusted their mitigation behaviors. Additionally, the relatively low carbon footprint at baseline may be partly explained by the fact that most participants were university students, who typically have lower diet‐ and transport‐related carbon footprints when institutional conditions support sustainable behavior (Cuy Castellanos et al., 2024; Ribeiro & Fonseca, 2022). For example, features of the university environment, such as access to public transport and restrictive parking policies, or the availability and affordability of plant‐based meals in the canteen, can encourage more environmentally sustainable transportation and dietary choices (Cuy Castellanos et al., 2024; Meier et al., 2022; Ribeiro & Fonseca, 2022). Testing the app in populations with higher baseline carbon footprints and different environmental constraints or opportunities would clarify whether the null findings for differences between the motivational and motivational + volitional app version are specific to this student sample or to the supportive context in which they live. Further, a formal in‐depth assessment of exposure to environmental facilitators and barriers in a more heterogeneous sample could provide a more ecological view of diet and transportation behaviors and additional insight into the intervention's efficacy.

Another factor that may explain the non‐significant group differences is the use of daily diaries to measure the mitigation behaviors in our study. Because self‐reporting on behavior is inherently a form of self‐monitoring, it may have contributed to modifying action control, which may have contributed to a decrease in carbon footprint. Our dietary data support this interpretation: Action control increased over the study period regardless of the intervention group and was linked to reduced diet‐related emissions. Similar self‐monitoring effects have been documented elsewhere, including healthy eating and physical activity (Michie et al., 2009) and reduced red‐meat consumption (Carfora et al., 2017). Future trials might include an intervention arm with self‐monitoring only and a no‐activity control arm to test the effects of self‐monitoring.

Regarding transportation‐related carbon footprint, we found no reduction over time. One potential explanation is that exposure to the transportation‐related intervention modules was optional and only a minority of participants set transportation‐related goals, which may have limited the overall intervention impact in this behavioral domain. In addition, transportation‐related behaviors are constrained by environmental and structural factors (i.e., the opportunities and barriers in the physical environment; Michie et al., 2011), such as availability of infrastructure (e.g., public transportation), and affordability of sustainable alternatives. For example, gasoline prices have been shown to moderate the effectiveness of interventions promoting low‐emission transport modes, with higher fuel costs increasing intervention success in contexts with greater access to public transport (Chevance et al., 2024). Such constraints may limit behavioral plasticity, suggesting that individual‐level behavioral interventions alone may have limited impact in this domain (Nisa et al., 2019). Consistent with this perspective, recent work has emphasized the potential relevance of structural or policy‐level factors for promoting changes in environmental behaviors (Albarracín et al., 2024).

### Targeted social‐cognitive determinants

The results showed that across participants, those with better action planning skills were more effective at reducing their diet‐related carbon footprint than those with lower action planning. This finding is consistent with evidence that action planning facilitates both mitigation and health behaviors (Kwasny et al., 2022; Sheeran et al., 2024). At the within‐person level, weeks characterized by higher action control were linked to a lower diet‐related carbon footprint, supporting the view that action control is a key behavioral determinant (Sniehotta et al., 2005). In weeks when participants reported higher coping planning than their average, their carbon footprint was higher the following week. Although unexpected, this pattern echoes earlier work showing negative associations between perceived coping planning and behavior (Inauen et al., 2018). A plausible explanation is that participants may have overestimated their coping skills or underestimated barriers, believing they were better prepared than they actually were, which could lead to less behavior change.

Taken together, these findings extend the well‐established relevance of HAPA‐related volitional variables from health behaviors (Zhang et al., 2019) to promoting a low‐emission diet. Along with the overall time effect observed in the study, they support the potential value of HAPA as a guiding framework for developing interventions aimed at reducing diet‐related carbon footprint. However, the lack of an inactive control group prevents firm conclusions about the framework's effectiveness. It is also noteworthy that although some intervention techniques central to our HAPA‐guided intervention are well suited for digital implementation, others, such as goal setting, may be more effective when delivered face‐to‐face (see Epton et al., 2017). Future research should explore the integration of digital and human‐delivered elements in a hybrid design as a potentially effective approach for developing HAPA‐guided interventions (Nahum‐Shani et al., 2022). In this direction, a further valuable approach to enhance the efficacy of theory‐driven pro‐environmental interventions is to more strongly integrate the needs and perspectives of target populations into intervention development, for instance, through qualitative approaches such as focus groups, co‐design sessions, or semi‐structured interviews (Yardley et al., 2015). This is particularly relevant in the present context, as awareness and engagement in mitigation behaviors vary considerably across countries and age groups (Boermans et al., 2024).

### Strengths and limitations

A key strength of this study was its randomized design. Further, the GROW intervention was grounded in a strong theoretical basis, addressing the lack of theoretical foundations in previous digital interventions aimed at supporting mitigation behaviors (Mosca et al., 2024). Additionally, even in the absence of an intervention effect, the analysis of the social‐cognitive determinants allowed us to explore potential drivers of mitigation behaviors and gain a better understanding of whether the intervention worked as intended. Furthermore, a novel aspect of this intervention was that we simultaneously targeted two high‐impact individual mitigation behaviors, dietary, and transportation behavior, with co‐benefits for both personal health and environmental sustainability. Nonetheless, future research including a single‐behavior intervention arm is needed to determine whether simultaneously addressing both mitigation behaviors is indeed an effective strategy. Moreover, future studies should formally investigate potential rebound effects of interventions targeting environmental co‐benefits (e.g., substituting car use with active transportation increases food‐related greenhouse gas emissions due to higher energy expenditure) (Chevance & Bernard, 2025). One final strength of the study lies in the transparent operationalization of the primary outcome using high‐quality, openly accessible emission factor datasets for both diet‐ and transportation‐related carbon footprints. This approach enabled ecologically valid translation of self‐reported behaviors into GHGE estimates, capturing variation both across and within behavior domains (e.g., meat vs. dairy; fish vs. seafood; car vs. train). By leveraging the SHARP Indicators Database (Mertens et al., 2019), we assessed daily consumption of meat and other animal‐based products (e.g., dairy and fish), reducing the risk of overlooking substitution effects (e.g., replacing one animal‐based product with another of equal or greater environmental impact) (Fresán et al., 2023). Nonetheless, in defining the food categories, we sought to balance detail with participant burden; as a result, some differentiation between items within the same category and their varying carbon footprints (e.g., fresh vs. hard cheese) may have been lost. Finally, the use of publicly available data sources enhances the reproducibility of our findings and supports the applicability of this method in future interventions targeting behavior‐related emissions.

This study is not without limitations. First, the primary outcome of the study, carbon footprint, was derived from self‐reported dietary and transportation behaviors. This introduces the possibility of reporting bias, including recall issues and social desirability. The former was minimized using electronic daily diaries (Stone et al., 2023); however, social desirability may still have influenced the results. Second, our results regarding transportation‐related and total carbon footprint are partly biased by the fact that engagement with the transportation‐related intervention module was optional, leading to limited intervention fidelity for this behavior. Although we accounted for engagement with this module by treating it as a covariate in our statistical models, this strategy cannot fully mitigate the biases. Nonetheless, as explained earlier, this decision was made to reduce participant burden during the weekly intervention modules and minimize potential dropout, which could have also impacted engagement with the diet‐related modules. Third, the absence of a no‐intervention control (e.g., a wait‐list group) limits our ability to determine whether the observed decline in diet‐related carbon footprint was attributable to the app content, possible reactivity of self‐monitoring, or simply due to participation in an intervention study. A fourth limitation concerns exclusion of individuals following a vegetarian or vegan diet. This assessment of participants' dietary status was based on self‐identification without providing a specific definition of dietary categories. This may have led to a conservative recruitment pool (e.g., some individuals following pescatarian diets may have identified as vegetarian and were therefore excluded from participation), which may have limited the representativeness of the sample. A further limitation concerns the additional informational material provided within the app to support engagement (see Table S6). Some of this content could be interpreted as offering *practical support* (BCT 3.2; Michie et al., 2013), which may introduce a degree of ambiguity regarding intervention differentiation, as these materials were accessible to both the motivational and motivational + volitional groups. Given that this content primarily targeted volitional processes, its inclusion across both groups could have slightly diluted potential group effects. However, any such influence is expected to be minimal, as these materials were optional, low in intensity, and mostly indirectly related to the two target behaviors. A final limitation relates to composition of our study sample, which was predominantly Western, Educated, Industrialized, Rich, and Democratic (WEIRD) and included a high prevalence of female participants (83%). This sex imbalance and demographic profile may limit the generalizability of the findings to more diverse populations. Similarly, because the study was conducted between mid‐April and mid‐August, its generalizability may not extend to colder seasons (e.g., fall or winter), which are typically associated with higher energy intake (Stelmach‐Mardas et al., 2016).

## CONCLUSIONS

This study investigated the efficacy of GROW, an app‐based intervention informed by the HAPA model, in promoting mitigation behaviors and reducing individual carbon footprints. Our findings did not support the hypothesis that targeting both the motivational and volitional phases of behavior change is more effective than a motivational‐only intervention. However, the reduction in diet‐related carbon footprint over the course of the study showed preliminary support for the efficacy of digital interventions for promoting a low‐emission diet. In this behavioral domain, the findings underscore the relevance of social‐cognitive factors such as action planning and action control and point to the value of the HAPA model as a theory‐based framework for informing future intervention development. In contrast, changing transportation behaviors through digital interventions appears to be less feasible, highlighting the need for additional interventions, e.g., those targeting structural factors.

## CONFLICT OF INTEREST STATEMENT

The authors declare no conflicts of interest.

## ETHICS STATEMENT

The ethics commission of the Faculty of Human Sciences at the University of Bern, Switzerland, approved this randomized parallel trial in January 2024.

## Supporting information


**Table S1.** CONSORT 2025 reporting checklist.
**Table S2.** TIDieR (Template for Intervention Description and Replication) checklist.
**Box S1.** Transportation‐related figures in the context of the study.
**Table S3.** Mapping between behavior change techniques (BCTs) and HAPA determinants.
**Table S4.** Implementation of behavior change techniques targeting dietary behavior change.
**Table S5.** Implementation of behavior change techniques targeting transportation behavior change.
**Table S6.** Content displayed to foster engagement with the app.
**Table S7.** Self‐report items for animal‐based food consumption.
**Figure S1.** Examples of displayed self‐report items for animal‐based food consumption in the GROW app.
**Box S2.** Calculation of greenhouse‐gas emissions (GHGE) of animal‐based food products per serving.
**Table S8.** Greenhouse‐gas emissions of animal‐based food categories per serving.
**Table S9.** Self‐report items for transportation behavior.
**Figure S2.** Examples of displayed self‐report items for transportation behavior in the GROW app.
**Table S10.** Greenhouse‐gas emissions of different transportation modes.
**Table S11.** Social‐cognitive determinants at baseline.
**Table S12.** Weekly participation in intervention modules.
**Table S13.** Sensitivity analysis: time, group, and time‐by‐group effects on individual carbon footprint with missing‐value imputation (Kalman filter).
**Table S14.** Sensitivity analysis: time, group, and time‐by‐group effects on individual carbon footprint with public‐holiday covariates.
**Table S15.** Time, group, and time‐by‐group effects on target social‐cognitive determinants.
**Table S16.** Time, group, and time‐by‐group effects on target social‐cognitive determinants with missing‐value imputation.
**Figure S3.** Diet‐related social‐cognitive determinants over time.
**Figure S4.** Transportation‐related social‐cognitive determinants over time.
**Table S17.** Multilevel model of social‐cognitive determinants on individual carbon footprint.

## Data Availability

Data and code can be found in the corresponding Open Science Framework project (https://osf.io/gakxm). The protocol and statistical analysis plan of the study were preregistered on April 3, 2024 (https://osf.io/bf594).
