# Supplementary material for: Theory‐based promotion of diet and transportation behavior change to reduce carbon footprint among students: Randomized parallel trial of the GROW app
Source: Appl Psychol Health Well Being. 2026 Jun 12;18(3):e70161. doi: 10.1111/aphw.70161 (PMC13262045; doi:10.1111/aphw.70161)
Supplement: Supplementary file 1 — Table S1. CONSORT 2025 reporting checklist. Table S2. TIDieR (Template for Intervention Description and Replication) checklist. Box S1. Transportation‐related figures in the context of the study. Table S3. Mapping between behavior change techniques (BCTs) and HAPA determinants. Table S4. Implementation of behavior change techniques targeting dietary behavior change. Table S5. Implementation of behavior change techniques targeting transportation behavior change. Table S6. Content displayed to foster engagement with the app. Table S7. Self‐report items for animal‐based food consumption. Figure S1. Examples of displayed self‐report items for animal‐based food consumption in the GROW app. Box S2. Calculation of greenhouse‐gas emissions (GHGE) of animal‐based food products per serving. Table S8. Greenhouse‐gas emissions of animal‐based food categories per serving. Table S9. Self‐report items for transportation behavior. Figure S2. Examples of displayed self‐report items for transportation behavior in the GROW app. Table S10. Greenhouse‐gas emissions of different transportation modes. Table S11. Social‐cognitive determinants at baseline. Table S12. Weekly participation in intervention modules. Table S13. Sensitivity analysis: time, group, and time‐by‐group effects on individual carbon footprint with missing‐value imputation (Kalman filter). Table S14. Sensitivity analysis: time, group, and time‐by‐group effects on individual carbon footprint with public‐holiday covariates. Table S15. Time, group, and time‐by‐group effects on target social‐cognitive determinants. Table S16. Time, group, and time‐by‐group effects on target social‐cognitive determinants with missing‐value imputation. Figure S3. Diet‐related social‐cognitive determinants over time. Figure S4. Transportation‐related social‐cognitive determinants over time. Table S17. Multilevel model of social‐cognitive determinants on individual carbon footprint. [file APHW-18-0-s001.docx]

Supplementary Materials to the Manuscript:

**Theory-based promotion of diet and transportation behavior change to reduce carbon footprint among students: Randomized parallel trial of the GROW app**

Authors:

Dario Baretta*, Carole Lynn Rüttimann*, & Jennifer Inauen

* These authors share first authorship

**Table of Content**

[Part 1. Supplementary Materials to the Methods Section of the Manuscript 3](#_Toc212564981)

[Table S1. CONSORT Checklist. 3](#_Toc212564982)

[Table S2. TIDieR Checklist. 6](#_Toc212564983)

[Box S1. Transportation-related Figures in the Context of the Study. 8](#_Toc212564984)

[Table S3. Mapping Between BCTs and HAPA Determinant. 9](#_Toc212564985)

[Table S4. Implementation of Behavior Change Techniques Targeting Dietary Behavior Change. 10](#_Toc212564986)

[Table S5. Implementation of Behavior Change Techniques Targeting Transportation Behavior Change. 17](#_Toc212564987)

[Table S6. Content Displayed to Foster Engagement with the App. 24](#_Toc212564988)

[Table S7. Self-report Items for Animal-Based Food Consumption. 30](#_Toc212564989)

[Figure S1. Examples of Displayed Self-report Items for Animal-Based Food Consumption in the GROW App. 33](#_Toc212564990)

[Box S2. Calculation of Greenhouse Gas Emissions (GHGE) of Animal-Based Food Products per Serving. 34](#_Toc212564991)

[Table S8. Greenhouse Gas Emissions of Animal-Based Food Categories per Serving. 35](#_Toc212564992)

[Table S9. Self-report Items for Transportation Behavior. 36](#_Toc212564993)

[Figure S2. Examples of Displayed Self-report Items for Transportation Behavior in the GROW App. 38](#_Toc212564994)

[Table S10. Greenhouse Gas Emissions of Different Transportation Modes. 39](#_Toc212564995)

[Part 2. Supplementary Materials to the Results Section of the Manuscript 40](#_Toc212564996)

[Table S11. Social-cognitive Determinants at Baseline. 40](#_Toc212564997)

[Table S12. Weekly Participation to Intervention Modules. 41](#_Toc212564998)

[Table S13. Sensitivity Analysis. Time, Group, and Time-by-group Effects on Individual Carbon Footprint With Missing Value Imputation (Kalman Filter). 42](#_Toc212564999)

[Table S14. Sensitivity Analysis. Time, Group, and Time-by-group Effects on Individual Carbon Footprint Including Weekend and Public Holidays as Covariate. 43](#_Toc212565000)

[Table S15. Time, Group, and Time-by-group Effects on Target Social-cognitive Determinants (No Missing Values Imputation). 45](#_Toc212565001)

[Table S16. Time, Group, and Time-by-group Effects on Target Social-cognitive Determinants with Missing Values Imputation (Last Observation Carried Forward). 47](#_Toc212565002)

[Figure S3. Diet-related Social-cognitive Determinants Over Time. 49](#_Toc212565003)

[Figure S4. Transportation-related Social-cognitive Determinants Over Time. 50](#_Toc212565004)

[Table S17. Multilevel Model of Social-cognitive Determinants on Individual Carbon Footprint. 51](#_Toc212565005)

[References 52](#_Toc212565006)

## Part 1. Supplementary Materials to the Methods Section of the Manuscript

### Table S1

### *CONSORT Checklist*

| Section/topic | No | CONSORT 2025 checklist item description | Reported on page no. |
| --- | --- | --- | --- |
| **Title and abstract** | | |  |
| Title and structured abstract | 1a | Identification as a randomised trial | 1 |
|  | 1b | Structured summary of the trial design, methods, results, and conclusions | 2 |
| **Open science** | | |  |
| Trial registration | 2 | Name of trial registry, identifying number (with URL) and date of registration | 1, 8 |
| Protocol and statistical analysis plan | 3 | Where the trial protocol and statistical analysis plan can be accessed | 8, 18 |
| Data sharing | 4 | Where and how the individual de-identified participant data (including data dictionary), statistical code and any other materials can be accessed | 1, 8 |
| Funding and conflicts of interest | 5a | Sources of funding and other support (eg, supply of drugs), and role of funders in the design, conduct, analysis and reporting of the trial | 1 |
|  | 5b | Financial and other conflicts of interest of the manuscript authors | 1 |
| **Introduction** | | |  |
| Background and rationale | 6 | Scientific background and rationale | 3-8 |
| Objectives | 7 | Specific objectives related to benefits and harms | N/A |

| **Methods** | | |  |
| --- | --- | --- | --- |
| Patient and public involvement | 8 | Details of patient or public involvement in the design, conduct and reporting of the trial | N/A |
| Trial design | 9 | Description of trial design including type of trial (eg, parallel group, crossover), allocation ratio, and framework (eg, superiority, equivalence, non-inferiority, exploratory) | 9 |
| Changes to trial protocol | 10 | Important changes to the trial after it commenced including any outcomes or analyses that were not prespecified, with reason | 17 |
| Trial setting | 11 | Settings (eg, community, hospital) and locations (eg, countries, sites) where the trial was conducted | 8, Supplementary Box S1 |
| Eligibility criteria | 12a | Eligibility criteria for participants | 9 |
|  | 12b | If applicable, eligibility criteria for sites and for individuals delivering the interventions (eg, surgeons, physiotherapists) | N/A |
| Intervention and comparator | 13 | Intervention and comparator with sufficient details to allow replication. If relevant, where additional materials describing the intervention and comparator (eg, intervention manual) can be accessed | 10-15, Supplementary Tables S3-S5 |
|  |  |  | **Reported on page no.** |
| Outcomes | 14 | Prespecified primary and secondary outcomes, including the specific measurement variable (eg, systolic blood pressure), analysis metric (eg, change from baseline, final value, time to event), method of aggregation (eg, median, proportion), and time point for each outcome | 11-15, Supplementary Tables S7-S10, , Figures S1-S2 |
| Harms | 15 | How harms were defined and assessed (eg, systematically, non-systematically) | N/A |
| Sample size | 16a | How sample size was determined, including all assumptions supporting the sample size calculation | 9 |
|  | 16b | Explanation of any interim analyses and stopping guidelines | N/A |
| Randomisation: |  |  |  |
| Sequence generation | 17a | Who generated the random allocation sequence and the method used | 15 |
|  | 17b | Type of randomisation and details of any restriction (eg, stratification, blocking and block size) | 8 |
|  |  |  |  |
| Allocation concealment mechanism | 18 | Mechanism used to implement the random allocation sequence (eg, central computer/telephone; sequentially numbered, opaque, sealed containers), describing any steps to conceal the sequence until interventions were assigned | 15 |
| Implementation | 19 | Whether the personnel who enrolled and those who assigned participants to the interventions had access to the random allocation sequence | 8, 15 |
| Blinding | 20a | Who was blinded after assignment to interventions (eg, participants, care providers, outcome assessors, data analysts) | 8, 15 |
|  | 20b | If blinded, how blinding was achieved and description of the similarity of interventions | 8-10, 15, 42 |
| Statistical methods | 21a | Statistical methods used to compare groups for primary and secondary outcomes, including harms | 16-18 |
|  | 21b | Definition of who is included in each analysis (eg, all randomised participants), and in which group | 18-19 |
|  | 21c | How missing data were handled in the analysis | 17-18 |
|  | 21d | Methods for any additional analyses (eg, subgroup and sensitivity analyses), distinguishing prespecified from post hoc | 17-18 |
| **Results** | | |  |
| Participant flow, including flow diagram | 22a | For each group, the numbers of participants who were randomly assigned, received intended intervention, and were analysed for the primary outcome | 19, 43 |
|  | 22b | For each group, losses and exclusions after randomisation, together with reasons | 18, 43 |
| Recruitment | 23a | Dates defining the periods of recruitment and follow-up for outcomes of benefits and harms | 18 |
|  | 23b | If relevant, why the trial ended or was stopped | N/A |
| Intervention and comparator delivery | 24a | Intervention and comparator as they were actually administered (eg, where appropriate, who delivered the intervention/comparator, how participants adhered, whether they were delivered as intended (fidelity)) | 19, Supplementary Table S12 |
|  | 24b | Concomitant care received during the trial for each group | N/A |
| Baseline data | 25 | A table showing baseline demographic and clinical characteristics for each group | 39-40 |
| Numbers analysed,  outcomes and estimation | 26 | For each primary and secondary outcome, by group:  ● the number of participants included in the analysis  ● the number of participants with available data at the outcome time point  ● result for each group, and the estimated effect size and its precision (such as 95% confidence interval)  ● for binary outcomes, presentation of both absolute and relative effect size | 19-21, 41, 44, Supplementary Tables S13-S17 |
| Harms | 27 | All harms or unintended events in each group | N/A |
| Ancillary analyses | 28 | Any other analyses performed, including subgroup and sensitivity analyses, distinguishing pre-specified from post hoc | Supplementary Tables S13-S17 |
| **Discussion** | | |  |
| Interpretation | 29 | Interpretation consistent with results, balancing benefits and harms, and considering other relevant evidence | 22-24 |
| Limitations | 30 | Trial limitations, addressing sources of potential bias, imprecision, generalisability, and, if relevant, multiplicity of analyses | 24-26 |

Citation: Hopewell S, Chan AW, Collins GS, Hróbjartsson A, Moher D, Schulz KF, et al. CONSORT 2025 Statement: updated guideline for reporting randomised trials. BMJ. 2025; 388:e081123. <https://dx.doi.org/10.1136/bmj-2024-081123>
© 2025 Hopewell et al. This is an Open Access article distributed under the terms of the Creative Commons Attribution License (<https://creativecommons.org/licenses/by/4.0/>), which permits unrestricted use, distribution, and reproduction in any medium, provided the original work is properly cited.

*We strongly recommend reading this statement in conjunction with the CONSORT 2025 Explanation and Elaboration and/or the CONSORT 2025 Expanded Checklist for important clarifications on all the items. We also recommend reading relevant CONSORT extensions. See [www.consort-spirit.org](http://www.consort-spirit.org).

### Table S2

### *TIDieR Checklist*
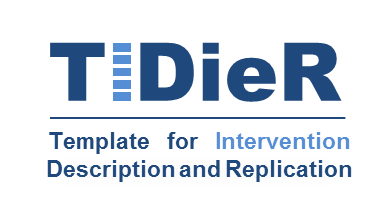


**The TIDieR (Template for Intervention Description and Replication) Checklist***

Information to include when describing an intervention and the location of the information

| **Item number** | **Item** | **Where located **** | |
| --- | --- | --- | --- |
|  |  | Primary paper  (page or appendix  number) | Other ^†^ (supplementary materials) |
|  | **BRIEF NAME** |  |  |
| **1.** | Provide the name or a phrase that describes the intervention. | 1 |  |
|  | **WHY** |  |  |
| **2.** | Describe any rationale, theory, or goal of the elements essential to the intervention. | 3–7 | 10 (Table S3) |
|  | **WHAT** |  |  |
| **3.** | Materials: Describe any physical or informational materials used in the intervention, including those provided to participants or used in intervention delivery or in training of intervention providers. Provide information on where the materials can be accessed (e.g. online appendix, URL). | 8–9 | 11–33 (Tables S4–S6) |
| **4.** | Procedures: Describe each of the procedures, activities, and/or processes used in the intervention, including any enabling or support activities. | 8–15 | 24–29 (Table S6) |
|  | **WHO PROVIDED** |  |  |
| **5.** | For each category of intervention provider (e.g. psychologist, nursing assistant), describe their expertise, background and any specific training given. | N/A | N/A |
|  | **HOW** |  |  |
| **6.** | Describe the modes of delivery (e.g. face-to-face or by some other mechanism, such as internet or telephone) of the intervention and whether it was provided individually or in a group. | 9-10 | N/A |
|  | **WHERE** |  |  |
| **7.** | Describe the type(s) of location(s) where the intervention occurred, including any necessary infrastructure or relevant features. | 8 | 9 (Box S1) |
|  | **WHEN and HOW MUCH** |  |  |
| **8.** | Describe the number of times the intervention was delivered and over what period of time including the number of sessions, their schedule, and their duration, intensity or dose. | 7–8, 42 (Figure 1) | 11–24 (Tables S4–S5) |
|  | **TAILORING** |  |  |
| **9.** | If the intervention was planned to be personalised, titrated or adapted, then describe what, why, when, and how. | 8 | 11–24 (Tables S4–S5) |
|  | **MODIFICATIONS** |  |  |
| **10.^ǂ^** | If the intervention was modified during the course of the study, describe the changes (what, why, when, and how). | 9 | 12 (note in Table S4) |
|  | **HOW WELL** |  |  |
| **11.** | Planned: If intervention adherence or fidelity was assessed, describe how and by whom, and if any strategies were used to maintain or improve fidelity, describe them. | 13 | 42 (Table S14) |
| **12.^ǂ^** | Actual: If intervention adherence or fidelity was assessed, describe the extent to which the intervention was delivered as planned. | 13, 43 (Figure 2) | 42 (Table S14) |

** **Authors** - use N/A if an item is not applicable for the intervention being described. **Reviewers** – use ‘?’ if information about the element is not reported/not sufficiently reported.

† If the information is not provided in the primary paper, give details of where this information is available. This may include locations such as a published protocol or other published papers (provide citation details) or a website (provide the URL).

ǂ If completing the TIDieR checklist for a protocol, these items are not relevant to the protocol and cannot be described until the study is complete.

* We strongly recommend using this checklist in conjunction with the TIDieR guide (see *BMJ* 2014;348:g1687) which contains an explanation and elaboration for each item.

* The focus of TIDieR is on reporting details of the intervention elements (and where relevant, comparison elements) of a study. Other elements and methodological features of studies are covered by other reporting statements and checklists and have not been duplicated as part of the TIDieR checklist. When a **randomised trial** is being reported, the TIDieR checklist should be used in conjunction with the CONSORT statement (see [www.consort-statement.org](http://www.consort-statement.org)) as an extension of **Item 5 of the CONSORT 2010 Statement.** When a **clinical trial** **protocol** is being reported, the TIDieR checklist should be used in conjunction with the SPIRIT statement as an extension of **Item 11 of the SPIRIT 2013 Statement** (see [www.spirit-statement.org](http://www.spirit-statement.org)). For alternate study designs, TIDieR can be used in conjunction with the appropriate checklist for that study design (see [www.equator-network.org](http://www.equator-network.org)).

### Box S1

### *Transportation-related Figures in the Context of the Study*

The study was conducted in Switzerland, a high-income country in Western Europe. Switzerland has one of the most efficient and dense public transport networks in Europe, integrating trains, buses, and trams at both regional and national levels. Among European countries, it has the highest share of passenger transport performed by public land transport (i.e., railways, coaches, and buses). Relative to its population size, the average distance travelled per inhabitant on national and international journeys is more than twice the European average (Eurostat, 2025). Nonetheless, in Switzerland, public land transport accounted for only 21% of total passenger kilometers by road and rail in 2023, with cars remaining the dominant mode of transport (69%) (Federal Statistical Office, 2025). Active transportation makes up a smaller share – cycling (2%) and walking (4%) – but is supported by well-developed cycling and pedestrian infrastructure. The promotion of cycling is also an important societal and political priority in Switzerland (Rérat & Ravalet, 2023).

### Table S3

### *Mapping Between BCTs and HAPA Determinants*

| **BCT** | **HAPA determinants** | **HAPA phases** | **Intervention groups** | |
| --- | --- | --- | --- | --- |
|  |  |  | **Motivational** | **Motivational + Volitional** |
| 1.1 Goal Setting (behavior) | - Intention | Motivational | ✔ | ✔ |
| 2.7 Feedback on Outcomes of Behavior | - Self-efficacy - Outcome expectancies | Motivational | ✔ | ✔ |
| 1.2 Problem Solving | - Coping planning - Self-efficacy | Volitional | ✘ | ✔ |
| 1.4 Action Planning | - Action planning | Volitional | ✘ | ✔ |
| 1.5 Review of Behavioral Goals | - Intention - Action control | Motivational/Volitional | ✘ | ✔ |
| 1.6 Discrepancy Between Current Behavior and Goal | - Action control - Self-efficacy | Volitional | ✘ | ✔ |
| 15.1 Verbal Persuasion About Capability | - Self-efficacy | Volitional | ✘ | ✔ |
| 3.3 Social Support (Emotional) | - Self-efficacy | Volitional | ✘ | ✔ |

*Note.* In this case the *outcome* of behavior is carbon footprint while the *behavior* is represented by diet and transportation (i.e., the mitigation behaviors).

### Table S4

### ***Implementation of Behavior Change Techniques Targeting Dietary Behavior Change***

| **Time point** | **Motivational group** | **Volitional group** | **BCT(s)** | **Implementation of the BCT(s)** |
| --- | --- | --- | --- | --- |
| Every day from T1 onwards | Yes | Yes | 2.7 Feedback on outcome of behavior | At T1 participants were introduced to a new page on the GROW app called “Progress.” This page displayed each participant’s diet-related carbon footprint (measured in kilograms of CO₂ equivalent) using two graphs: Graph 1 showed their weekly progress, and Graph 2 showed their daily progress. The graphs were updated each time participants reported their daily consumption of animal-based products. |
| T1 | Yes | Yes | 1.1 Goal setting (behavior) | *Apart from being based on behavior, goals should also be* ***challenging but feasible*** *at the same time. Challenging in a sense that the goal pushes your limits and feasible in a way that you can have several successful experiences in a row which help you stay motivated.  Let’s make an example: If Lina's goal is to introduce meat free days for 6 days a week, this might be challenging but not realistic for her. With a lot of effort, she might accomplish 5 meat free days in a week, which is a lot, but she wouldn’t reach her goal. On the other hand, if Lina aims for just 1 meat free day a week, this might be too easy for her and also would not reduce her carbon footprint by much. Therefore, Lina should try to find his individual sweet spot between difficult but realistic goals.* |
| T1 | Yes | Yes | 1.1 Goal setting (behavior) | *Now, let’s set your goal(s) for the upcoming week! We encourage you to set a behavioral goal for your dietary behavior as well as one for your travel behavior. Remember to set goals that force you out of your comfort zone but are realistic at the same time.  First, choose a* ***dietary goal*** *you would like to pursue during the upcoming week from the following list. We encourage you to aim for one of the first 3 goals since they are more challenging. If you think that none of them are feasible for you, choose one of the last 3 options. [single choice] o Meat free day(s) o Dairy free day(s) o Animal product free day(s) o Meat free meal(s) o Dairy free meal(s) o Animal product free meal(s)  Then, to make your dietary/travel goal(s) more specific, let's define how many times in the upcoming week you would like to pursue it. [single choice] o 1 o 2 o 3 o 4 o 5 o 6  o 7* |
| T2,T3, T4 | Yes | Yes | 1.1 Goal setting (behavior) | *First, choose a dietary goal you would like to pursue during the upcoming week from the following list. We encourage you to aim for one of the first 3 goals since they are more challenging. If you think that none of them are feasible for you, choose one of the last 3 options. If you would like, you can also choose multiple dietary goals. [single choice] o Meat free day(s) o Dairy free day(s) o Animal product free day(s) o Meat free meal(s) o Dairy free meal(s) o Animal product free meal(s)   Then, to make your dietary goal(s) more specific, let's define how many times in the upcoming week you would like to pursue it. [single choice] o 1 o 2 o 3 o 4 o 5 o 6  o 7 6* |
| T1 | No | Yes | 1.4 Action planning | *An effective strategy to successfully implement your goal is to create an* ***action plan****. In an action plan you note very precisely what you have to do in a specific situation. Let’s look at an example: Each morning when Lina arrives at the university, she first goes to the cafeteria and buys a chocolate bread for breakfast. However, Lina’s goal is to have at least 3 dairy free meals per week. She chooses to change her breakfast habit and therefore formulates the following action plan: «WHEN* ***I go to the cafeteria in the morning****, THEN* ***I buy a vegan Birchermüesli****.»  Let’s go! Create a fitting action plan for your dietary goal, same as Lina did. Please make sure to formulate at least one action plan to implement your goal. Depending on your goal and the situations you encounter in your daily life, you might even find having more than one action plan more suitable. Be aware that your action plans need to be formulated as in the example above. Therefore, you only need to enter your own version of the text that is written in* ***bold text****.   Action plan 1 WHEN: ___________ THEN: ___________  Action plan 2 (optional) WHEN: ___________ THEN: ___________  Action plan 3 (optional) WHEN: ___________ THEN: ___________  Amazing! You have chosen your dietary goal and written down your first action plan(s). Let’s have a look at it:* ***Your dietary goal:*** *«My dietary goal for next week is to have [display frequency with embedded data] [display goal with embedded data].* ***Your action plan(s):*** *«When [display with embedded data], then [display with embedded data].» After finishing this reflection day, you will be able to revisit your goal(s) and action plan(s) in the Progress page of the app.   This is your last chance to change your action plan(s). Afterwards your action plans stay like this for the upcoming week, and you won’t be able to make any changes. In case you would like to adjust your action plan(s), go back to the previous step. If your happy with your entries, please continue.* |
| T2, T3, T4 | No | Yes | 1.4 Action planning | *Now it's time to create one or several* ***action plans*** *in order to specify how you plan to implement your dietary goal. Please make sure to formulate at least one action plan to implement your goal. Depending on your goal(s) and the situations you encounter in your daily life, you might even find having more than one action plan more suitable.   Action plan 1 WHEN: ___________ THEN: ___________  Action plan 2 (optional) WHEN: ___________ THEN: ___________  Action plan 3 (optional) WHEN: ___________ THEN: ___________  Amazing! You have chosen your dietary goal and written down your first action plan(s). Let’s have a look at it:* ***Your dietary goal::*** *«My dietary goal for next week is to have [display frequency with embedded data] [display goal with embedded data].* ***Your action plan(s):*** *«When [display with embedded data], then [display with embedded data].» After finishing this reflection day, you will be able to revisit your goal(s) and action plan(s) in the Progress page of the app.   This is your last chance to change your action plan(s). Afterwards your action plans stay like this for the upcoming week, and you won’t be able to make any changes. In case you would like to adjust your action plan(s), go back to the previous step. If your happy with your entries, please continue.* |
| T2, T3, T4 | No | Yes | 1.6 Discrepancy between current behavior and goal  with ***15.1 Verbal persuasion about capability*** | *It’s time again to reflect! One week ago, we asked you to set behavioral goals regarding diet and optionally also regarding travel. Let’s have a look at your performance over the last week in regard of these goals.    Your* ***dietary goal*** *of last week was: [display times chosen from embedded data] [display chosen goal from embedded data]  Did you reach last week’s dietary goal? [single choice] o Yes [go to page A] o No [go to page B] o Partially (e.g., you set your goal for 2 days and implemented it successfully on 1 day but failed to implement it a 2nd time) [go to page C]    [Page A] Well done,* ****we are confident you will be able to keep this up in the coming weeks!**** *Did you stick to your action plan(s) or did you implement your dietary goal differently?  Your action plan(s) for the past week: [display WHEN-THEN plan from embedded data]  o I implemented my goal as I had planned.*  *o I implemented my goal differently than I had planned.   [Page B]* ****You are not quite there yet but we are confident that you will accomplish your goal(s) in the future!**** *[Page C] This is already quite something!* ****We are confident that you will fully reach your dietary goal on your next try.**** |
| T2, T3, T4 | No | Yes | 1.5 Review of behavioral goals | *So, you [embedded data: did / did not / did partially ] reach your dietary goal.    Remember to set* ***challenging but feasible*** *goals. If your dietary goal for last week was too challenging, think about choosing an easier goal for the upcoming week. If your dietary goal was too easy, think about choosing a more challenging option this time.* |
| T2, T3, T4 | Yes | Yes | 1.1 Goal setting | *Now, let’s set your* ***dietary goal*** *for the upcoming week! You can choose if you would like to stick to the same goal you set last week, meaning that you would set a goal for the same behavior (e. g., meat free days) and the same number of days (e. g., for 3 days). However, you can also choose another goal for the upcoming week (this also includes choosing the same behavior but targeting another number of days). [single choice] o stick to the same goal I set last week  o change my goal* |
| T3 | No | Yes | 1.2 Problem solving | *You have now been part of this study for three weeks and working on reducing your carbon footprint for the past two weeks. Whether you achieved your goal or not, whilst using GROW, you have most likely encountered barriers to implementing your dietary goals. Maybe you were able to overcome those barriers or maybe you did not achieve your goals because of them. Let’s make an example of what a barrier might look like: Lina’s dietary goal was to introduce 2 meet free days into your week. She chose to implement them on Tuesday and Sunday. So, she formulated her action plan as follows: «When it is a Sunday or Tuesday, then I do not eat meat.» Each Sunday, Lina visits her parents for dinner. This time, her parents prepared spaghetti carbonara. She realized she forgot to mention to her parents that she planned on eating vegetarian this Sunday. So, Lina just encountered a barrier to implementing her dietary goal.    Please think about the barriers you could be challenged with when pursuing your dietary goal(s) in the upcoming week. Are they similar to the barrier Lina encountered? Note all the barriers you can think of. It's important to describe these problems as specific as possible: ____________ large text field]   Great! Now that you are aware of your barriers, let’s work on how you can overcome them. There are two ways how one can work around such barriers: either with problem solving or coping planning. First, let’s have a look at* ***problem solving****. Problem solving is a strategy that helps you find an immediate solution to a barrier you are currently encountering, so you can still reach your goal according to your action plan(s). In Lina’s case this might be eating the spaghetti without the carbonara sauce her parents prepared. Now, it’s your turn. Try to think of specific problem-solving strategies to immediately overcome the barriers you noted down earlier. As a first step, you already recorded the following barriers: [insert embedded data here]. Please choose one difficulty that you’ve written down.  As a second step, please write down different solutions to overcome this barrier. Try to think of as many alternative solutions as possible. For this step it’s important not to rate any solutions! This means, write down absolutely everything that comes to mind.  __________ [large text field]    As a third step you must decide on one solution. Which solution fits you and the situation the best? Which solution is easiest to implement? What could you take care of right now? In the end you choose the solution that works best for you. Please write down which of the solutions you choose of the ones you priorly came up with?   __________ [ text field]   Well done! Whenever you encounter a barrier for implementing your dietary goal throughout the upcoming week, try to think of and make use of your solution: [display with embedded data]. After finishing the reflection day, you can find the solution you just formulated in the Progress page of the app.   As mentioned earlier, you can also overcome your barriers by using* ***coping planning****. The idea behind coping planning is to implement your goal in a different way than you had planned. In our example, Lina did not manage to have a meat free Sunday because she forgot to tell her parents about her new dietary plans. A coping plan for such a situation could be to just adapt the action plan and postpone her meat free day to Monday instead of having a meat free Sunday. This way, Lina can still reach her goal.* |
| T4 | No | Yes | 1.2 Problem solving with ***15.1 Verbal persuasion about capability*** | *Last week, we learned about barriers to achieving goals. Did you encounter any such barriers to achieving your dietary goal during the last week? o Yes [continue] o No [skip this whole part]  Please note the barriers and problems you encountered down here. Make sure to mention them even if you managed to overcome them: __________ [large text field]   Were you able to overcome these barriers?  o Yes [show page A] o No [show page B]  [Page A]  Well done,* ****we are confident you will be able to keep this up in the coming weeks!**** *Which specific strategies did you use to overcome the barriers you mentioned in regard to your dietary goal(s)? Please note them down here: ____________   [Page B]* ****We are confident that by reflecting on potential problem solving and coping planning strategies, you will be able to overcome these barriers next week!**** *Let's work on a strategy together. First, let's start with* ***problem solving****. Problem solving is a strategy that helps you find an immediate solution to a barrier you are currently encountering, so you can still reach your goal according to your action plan(s).  As a first step, you already recorded the following barriers: [insert embedded data here]. Please choose one difficulty you would like to work of those you have written down earlier: __________ [ text field].   As a second step, please write down different solutions to overcome this barrier. Try to think of as many alternative solutions as possible. For this step it’s important not to rate any solutions! This means, write down absolutely everything that comes to mind.  __________ [large text field]   As a third step you must decide on one solution. Which solution fits you and the situation the best? Which solution is easiest to implement? What could you take care of right now? In the end you choose the solution that works best for you. Please write down which of the solutions you choose of the ones you priorly came up with?   __________ [text field]   As you know, you can also overcome your barriers by using* ***coping planning****. The idea behind coping planning is to implement your goal in a different way than you had planned. Let's look at our example from last week: Lina’s dietary goal was to introduce 2 meet free days into your week. She chose to implement them on Tuesday and Sunday. So, she formulated her action plan as follows: «When it is a Sunday or Tuesday, then I do not eat meat.» Each Sunday, Lina visits her parents for dinner. This time, her parents prepared spaghetti carbonara. She realized she forgot to mention to her parents that she planned on eating vegetarian this Sunday. So, Lina just encountered a barrier to implementing her dietary goal. Lina decided to eat the carbonara sauce anyway since she had already prepared a coping plan for a barrier like this. Her plan was to switch days and postpone her meat free day to Monday instead of having a meat free Sunday. This way, Lina can still reach her goal. What could be a suitable coping plan to overcome your difficulty? __________ [ text field]  Well done! Whenever you encounter a barrier for implementing your dietary goal(s) throughout the upcoming week, try to think of and make use of the strategies you wrote down today.* ***Problem solving strategy:*** *[display with embedded data].* ***Coping plan:*** *[display with embedded data].  After finishing the reflection day, you can find the solutions you just formulated in the Progress page of the app.* |

*Note.* BCTs = Behavior Change Techniques. BCTs are named following the Behavior Change Technique Taxonomy (Michie et al., 2013). The BCT 15.1, Verbal persuasion about capability, is highlighted in blue since it was presented integrated with other BCTs.

### Table S5

### ***Implementation of Behavior Change Techniques Targeting Transportation Behavior Change***

| **Time point** | **Motiva-tional group** | **Volitional group** | **BCT(s)** | **Implementation of the BCT(s)** |
| --- | --- | --- | --- | --- |
| Every day from T1 onwards | Yes | Yes | 2.7 Feedback on outcome of behavior | At T1 participants were introduced to a new page on the GROW app called “Progress.” This page displayed the participant’s transportation-related carbon footprint (measured in kilograms of CO₂ equivalent) using two graphs: Graph 1 showed their weekly progress, and Graph 2 showed their daily progress. The graphs were updated each time the participant reported their daily transportation behavior. |
| T1 | Yes | Yes | 1.1 Goal setting (behavior) | *Now, you can choose if you would also like to set a* ***travel goal*** *for the upcoming week. [single choice] o I am motivated to set a travel goal! o Pursuing a dietary goal is sufficient for me.   Please choose or write down, for which of your journeys you would like to change your travel behavior. In the following list, we prepared a few examples. If none of those apply to you or you would prefer another journey, please formulate a travel goal yourself. Always remember to set challenging but feasible goals. [single choice]  o from home to the university and back o from home to work and back o from home to the supermarket and back o from home to the gym and back o formulate own travel goal: ________   I would like to replace my usual way of transportation for the chosen journey by... [single choice] o public transportation o bike o E-bike o foot o other: ________  Then, to make your travel goal(s) more specific, let's define how many times in the upcoming week you would like to pursue it. [single choice] o 1 o 2 o 3 o 4 o 5 o 6  o 7* |
| T2, T3, T4 | Yes | Yes | 1.1 Goal setting (behavior) | *Please choose or write down, for which of your journeys you would like to change your travel behavior. In the following list, we prepared a few examples. If none of those apply to you or you would prefer another journey, please formulate a travel goal yourself. If you would like, you can also choose multiple travel goals. [single choice] o from home to the university and back o from home to work and back o from home to the supermarket and back o from home to the gym and back o formulate own travel goal: ________   I would like to replace my usual way of transportation for the chosen journey by... [multiple choice] o public transportation o bike o E-bike o foot o other: ________  Then, to make your travel goal(s) more specific, let's define how many times in the upcoming week you would like to pursue it. [single choice] o 1 o 2 o 3 o 4 o 5 o 6  o 7* |
| T1 | No | Yes | 1.4 Action planning | *As you know by now,* ***action plans*** *can be an effective strategy to successfully implement your goal. Let’s look at an example: Each morning when Leo goes at the university, he takes the bus. However, Leo's goal is to replace his usual way of traveling to the university and back by taking the bike. Therefore, he formulates the following action plan: «WHEN* ***I go to the university****, THEN* ***I take my bike****.»  Now it's your turn! Create a fitting action plan for your travel goal, same as Leo did. Please make sure to formulate at least one action plan to implement your goal. Depending on your goal and the situations you encounter in your daily life, you might even find having more than one action plan more suitable. Be aware that your action plans need to be formulated as in the example above. Therefore, you only need to enter your own version of the text that is written in* ***bold text****.   Action plan 1 WHEN: ___________ THEN: ___________  Action plan 2 (optional) WHEN: ___________ THEN: ___________  Action plan 3 (optional) WHEN: ___________ THEN: ___________  Amazing! You have chosen your travel goal and written down your first action plan(s). Let’s have a look at it:* ***Your travel goal:*** *«My travel goal for next week is to have [display frequency with embedded data] [display goal with embedded data].* ***Your action plan(s):*** *«When [display with embedded data], then [display with embedded data].» After finishing this reflection day, you will be able to revisit your goal(s) and action plan(s) in the Progress page of the app.   This is your last chance to change your action plan(s). Afterwards your action plans stay like this for the upcoming week, and you won’t be able to make any changes. In case you would like to adjust your action plan(s), go back to the previous step. If your happy with your entries, please continue.* |
| T2, T3, T4 | No | Yes | 1.4 Action planning | *Now it's time to create one or several* ***action plans*** *in order to specify how you plan to implement your travel goal. Please make sure to formulate at least one action plan to implement your goal. Depending on your goal(s) and the situations you encounter in your daily life, you might even find having more than one action plan more suitable.   Action plan 1 WHEN: ___________ THEN: ___________  Action plan 2 (optional) WHEN: ___________ THEN: ___________  Action plan 3 (optional) WHEN: ___________ THEN: ___________  Amazing! You have chosen your travel goal and written down your first action plan(s). Let’s have a look at it:* ***Your travel goal:*** *«My travel goal for next week is to have [display frequency with embedded data] [display goal with embedded data].* ***Your action plan(s):*** *«When [display with embedded data], then [display with embedded data].» After finishing this reflection day, you will be able to revisit your goal(s) and action plan(s) in the Progress page of the app.   This is your last chance to change your action plan(s). Afterwards your action plans stay like this for the upcoming week, and you won’t be able to make any changes. In case you would like to adjust your action plan(s), go back to the previous step. If your happy with your entries, please continue.* |
| T2, T3, T4 | No | Yes | 1.6 Discrepancy between current behavior and goal  with ***15.1 Verbal persuasion about capability*** | *Did you set a travel goal for last week? o Yes [go to page A] o No [skip to the end of the survey]*  *Great, we are half-way through! Now, let’s have a look at last week’s* ***travel goal****.    Your travel goal of last week was: [display times chosen from embedded data] [display chosen goal from embedded data]  Did you reach last week’s travel goal? [single choice] o Yes [go to page B] o No [go to page C] o Partially (e.g., you set your goal for 2 days and implemented it successfully on 1 day but failed to implement it a 2nd time) [go to page D]  o I did not set a travel goal [skip the next part]    [Page B] Well done,* ****we are confident you will be able to keep this up in the coming weeks!**** *Did you stick to your action plan(s) or did you implement your dietary goal differently?  Your action plan(s) for the past week: [display WHEN-THEN plan from embedded data]  o I implemented my goal as I had planned. o I implemented my goal differently than I had planned.    [Page C]* ****You are not quite there yet but we are confident that you will accomplish your goal(s) in the future!**** *[Page D] This is already quite something!* ****We are confident that you will fully reach your dietary goal on your next try.**** |
| T2, T3, T4 | No | Yes | 1.5 Review of behavioral goals | *So, you [embedded data: did / did not / did partially ] reach your travel goal.    Remember to set* ***challenging but feasible*** *goals. If your travel goal for last week was too challenging, think about choosing an easier goal for the upcoming week. If your travel goal was too easy, think about choosing a more challenging option this time.* |
| T2, T3, T4 | Yes | Yes | 1.1 Goal setting | *Would you also like to set a* ***travel goal f****or the upcoming week? [single choice] o Yes, I am motivated to set a travel goal [continue] o Pursuing a dietary goal is sufficient for me [skip the next part]   Then, let’s set your travel goal for the upcoming week! You can choose if you would like to stick to the same goal you set last week, meaning that you would set a goal for the same behavior (e. g., meat free days) and the same number of days (e. g., for 3 days). However, you can also choose another goal for the upcoming week (this also includes choosing the same behavior but targeting another number of days). [single choice] o stick to the same goal I set last week  o change my goal* |
| T3 | No | Yes | 1.2 Problem solving | *Whether you achieved last week’s goal or not, you have most likely encountered barriers to implementing your travel goals. Maybe you were able to overcome those barriers or maybe you did not achieve your goals because of them. Let’s make an example of what a barrier to reaching your travel goals might look like: Leo’s travel goal was to perform 3 active transportation days per week. He planned to implement them on Monday, Tuesday, and Friday by going to university by bike instead of by bus. On Tuesday, it rained heavily. So right then, Leo encountered a barrier to implementing his travel goal.    Please think about the barriers you could be challenged with when pursuing your travel goal(s) in the upcoming week. Are they similar to the barrier Leo encountered? Note all the barriers you can think of. It's important to describe these problems as specific as possible: ____________ large text field]   Great! Now that you are aware of your barriers, let’s work on how you can overcome them. As you learned earlier, there are two ways how one can work around such barriers. In Leo’s case, an example for* ***problem solving*** *could be as follows: Leo could use a raincoat and rain pants, so he can still perform his planned active transportation day on Tuesday. Now, it’s your turn. Try to think of specific problem solving strategies to immediately overcome the barriers you noted down earlier. As a first step, you already recorded the following barriers: [insert embedded data here]. Please choose one difficulty that you’ve written down.  As a second step, please write down different solutions to overcome this barrier. Try to think of as many alternative solutions as possible. For this step it’s important not to rate any solutions! This means, write down absolutely everything that comes to mind.  __________ [large text field]    As a third step you must decide on one solution. Which solution fits you and the situation the best? Which solution is easiest to implement? What could you take care of right now? In the end you choose the solution that works best for you. Please write down which of the solutions you choose of the ones you priorly came up with?   __________ [ text field]   Well done! Whenever you encounter a barrier for implementing your dietary goal throughout the upcoming week, try to think of and make use of your solution: [display with embedded data]. After finishing the reflection day, you can find the solution you just formulated in the Progress page of the app.   As mentioned earlier, another way to cope with barriers is to make use of the concept of* ***coping planning****. In Leo’s case this might be postponing his active transportation day to another day later this week.* |
| T4 | No | Yes | 1.2 Problem solving with ***15.1 Verbal persuasion about capability*** | *Last week, we learned about barriers to achieving goals. Did you encounter any such barriers to achieving your travel goal during the last week? o Yes [continue] o No [skip this whole part]*  *Please note the barriers and problems you encountered down here. Make sure to mention them even if you managed to overcome them: __________ [large text field]   Were you able to overcome these barriers?  o Yes [show page A] o No [show page B]  [Page A]  Well done,* ****we are confident you will be able to keep this up in the coming weeks!**** *Which specific strategies did you use to overcome the barriers you mentioned in regard to your dietary goal(s)? Please note them down here: ____________   [Page B]* ****We are confident that by reflecting on potential problem solving and coping planning strategies, you will be able to overcome these barriers next week!**** *Let's work on a strategy together. First, let's start with* ***problem solving****. Problem solving is a strategy that helps you find an immediate solution to a barrier you are currently encountering, so you can still reach your goal according to your action plan(s).  As a first step, you already recorded the following barriers: [insert embedded data here]. Please choose one difficulty you would like to work of those you’ve written down earlier: __________ [ text field].   As a second step, please write down different solutions to overcome this barrier. Try to think of as many alternative solutions as possible. For this step it’s important not to rate any solutions! This means, write down absolutely everything that comes to mind.  __________ [large text field]   As a third step you must decide on one solution. Which solution fits you and the situation the best? Which solution is easiest to implement? What could you take care of right now? In the end you choose the solution that works best for you. Please write down which of the solutions you choose of the ones you priorly came up with?   __________ [text field]   As you know, you can also overcome your barriers by using* ***coping planning****. The idea behind coping planning is to implement your goal in a different way than you had planned. Let's look at our example from last week: Leo’s travel goal was to perform 3 active transportation days per week. He planned to implement them on Monday, Tuesday, and Friday by going to university by bike instead of by bus. On Tuesday, it rained heavily. So right then, Leo encountered a barrier to implementing his travel goal. In Leo’s case a coping plan might be checking his weather app and postponing his active transportation day to another, less rainy day later this week. This way, Leo can still reach his goal. What could be a suitable coping plan to overcome your difficulty? __________ [ text field]  Well done! Whenever you encounter a barrier for implementing your dietary goal(s) throughout the upcoming week, try to think of and make use of the strategies you wrote down today.* ***Problem solving strategy:*** *[display with embedded data].* ***Coping plan:*** *[display with embedded data].  After finishing the reflection day, you can find the solutions you just formulated in the Progress page of the app.* |

*Note.* BCTs = Behavior Change Techniques. BCTs are named following the Behavior Change Technique Taxonomy (Michie et al., 2013). The BCT 15.1, Verbal persuasion about capability, is highlighted in blue since it was presented integrated with other BCTs.

### Table S6

### ***Content Displayed to Foster Engagement with the App***

|  | **Day** | **Content** |
| --- | --- | --- |
| **Tips** | | |
|  | 2 | **Vegan cream cheese DIY** The production of dairy products burdens our environment. Plant-based alternatives are not CO2-neutral but still have a significantly better environmental footprint. Here's a recipe for vegan cream cheese. Give it a try!  • 200g cashew nuts, 1 lemon, a handful of parsley, apple cider vinegar, water • Soak cashews in water for 12 hours • When soft, blend cashews in a mixer, add parsley, vinegar, and juice of half a lemon • Puree until creamy and add salt and pepper to taste • Chill for an hour and serve with fresh bread |
|  | 7 | **4 tips for more environmentally friendly meat consumption** 1. Consume more consciously and less often 2. Eat more chicken, less pork, and especially less beef (which has the highest CO2 footprint) 3. If you do have beef, choose local meat from grass-fed cows instead of Angus steaks from South America. But no 4. matter what type of meat, local is always better for the environment! Reduce the pressure – start slowly and set realistic goals, but stick with it :) |
|  | 8 | **Bikesharing** Use bike sharing instead of getting into your car. Bike sharing is the ideal addition to private and public transport for short distances. In the city center, you can reach your destination faster by bike and reduce motorized traffic in the center. You can easily use an app that tells you where the nearest bike station is and how many bikes are available. There are different rates to choose from. If you don’t know it yet, take a look at: https://www.publibike.ch/de/publibike/pricing |
|  | 10 | **Vegan bakeries and restaurants in Bern** You want to go out for a meal or breakfast, but you're not sure where to find vegan food? We can help you out!  • Vegan Bakery: BakeryBakery https://www.bakerybakery.ch • Vegan Café: Tingel Kringel, Beans and Nuts • Vegan Restaurants: Energy Kitchen, Vabakkam, Colorz Kitchen, Swing Kitchen, Tibits, and many more are either fully vegan or offer a selection of vegan dishes.  More details on the blog: https://www.baerner-meitschi.ch/die-besten-veganen-restaurants-in-bern/ |
|  | 13 | **Seasonal calendar for fruits and vegetables** Fruits and vegetables are most environmentally friendly when they are grown and harvested during their natural season and not transported for long periods before being consumed. Did you know that there are seasonal fruit and vegetable calendars that show you when different fruits or vegetables are in season? You can find them for download here: https://fooby.ch/en/cookery-school/seasonal-calendar.html?startAuto1=0  The calendar is also perfect for printing and hanging in your kitchen. That way, you’ll always know which foods are in season. |
|  | 15 | **Four sources of non-animal protein** • Legumes • Tofu • Quinoa • Nuts |
|  | 17 | **Foodsharing – share and save money!**  Food Sharing is a practice where individuals or groups commit to sharing food instead of wasting it. Did you know that food 'distributors' are set up near you, where you can drop off food you no longer want or need? You can leave food in a distributor near you and pick up food others no longer want – and even save some money. Here you can find maps showing the nearest distributors:  • https://foodsharing.network • https://www.madamefrigo.ch/de/ |
|  | 19 | **Identifying processed foods** Processed foods are often far from environmentally friendly (and naturally also not healthy) due to their production, ingredients, and packaging. Here are some tips on how to recognize them best: Check the ingredients list:  • Artificial ingredients? • Lots of refined carbohydrates? • Low in nutrients and fiber? • High sugar content? • Trans fats and vegetable oils? = Processed foods! |
|  | 22 | **Making your own oat milk? No problem!** Whether your local supermarket is out of Oatly, or you simply want to control the ingredients in the milk you pour over your muesli – making your own oat milk is often the best and healthiest choice! Oat milk is one of the easiest vegan milk alternatives to make. Unlike most nut milks, you don’t need a special bag – a simple sieve will do the job. Which type of oats is best for oat milk? Use old-fashioned rolled oats, as quick oats or steel-cut oats will result in a more watery consistency. Try this creamy, lightly sweet homemade oat milk recipe from The Green Creator: https://youtu.be/d9b3JS4eIRI  By the way, oat milk is the plant-based milk alternative with the best CO2 footprint. |
|  | 25 | **Carpooling** For longer trips, use the carpooling principle instead of driving alone! Did you know that the website blablacar.de is the world’s leading community-based travel network, enabling over 100 million people in 22 countries to share a car ride? The eco-friendly and people-friendly carpooling mobility network saves 1.6 million tons of CO2 and enables 120 million human connections each year. Carpooling doubles the occupancy of cars while operating a carbon-saving network. You can either offer or join a ride: https://www.blablacar.de |
|  | 26 | **The relevance of regional products** Due to transport by truck, ship, or even airplane, the carbon footprint of many foods is extremely poor. Sure, mangoes and pineapples don’t grow in Switzerland, so they need to be imported. But maybe you’d like to opt for less exotic fruits or other imported foods. Instead, choose regional fruits and vegetables. This is easy for many types, such as strawberries, tomatoes, or apples. Nowadays, packaged and unpackaged foods are labeled so you can recognize the country of origin. While strolling through the farmers' market, you can shop from producers in your region. In many cities, there are even collection services now – you order online and pick up the regional products at a collection point from various providers, or have them delivered to your home. This way, you can discover new seasonal vegetables, and the included recipes show you how to prepare them. Here are some of these services for the area of Bern:  • https://xn--gmesgarte-r9a.ch/index.html  • https://oepfelchasper.ch/de/ • https://ruedu.ch/ |
|  | 29 | **To good to go – Food save** Too good to go – saving food Every day, delicious, fresh food ends up in cafes, restaurants, hotels, stores, and from producers in the trash – just because it wasn’t sold in time. With the Too Good To Go app, customers can buy and pick up this food at a lower price. Here’s the app:  • Playstore: https://play.google.com/store/apps/details?id=com.app.tgtg&hl=de_CH&gl=US • App Store: https://apps.apple.com/ch/app/too-good-to-go/id1060683933 |
|  | 32 | **Still eating fish, but want to make your consumption more sustainable?** • Trout from local waters • Carp • Herring from Norway |
|  | 35 | **Vegan recipes, but where?** Have you ever stood in front of your fridge and wondered what to cook next? If so, we have three great websites with many plant-based but simple recipes. Check them out, they might help spice up your cooking skills:  • https://eat-this.org • https://www.zuckerjagdwurst.com/de • https://serayi.com/rezepte/ **•** https://www.kptncook.com/de/index |
| **Short readings** | | |
|  | 3 | **What are greenhouse gas emissions?** Greenhouse gas emissions include carbon dioxide and methane. They are produced by burning fossil fuels like gasoline for driving or coal for heating a building. For example, clearing land and forests can also release carbon dioxide. Energy, industry, agriculture, and waste disposal are among the largest contributors to greenhouse gas emissions. Looking back 2 million years, it becomes clear that greenhouse gas concentrations today are at their highest levels and are continuing to rise. As a result, the Earth is about 1.1°C warmer than it was in 1800. For more information see: https://www.un.org/en/climatechange/science/key-findings |
|  | 4 | **Zoonoses** The term "zoonosis" originates from the Greek words zoon (living being) and nosos (disease). Zoonoses are infectious diseases caused by bacteria, parasites, fungi, prions (abnormal proteins), or viruses that can be transmitted back and forth between animals and humans. According to a study published in Nature, 60% of so-called emerging infectious diseases have a zoonotic origin. The majority of these zoonoses, over 70%, come from wildlife. Zoonoses also include diseases transmitted by domestic animals such as pigs, cows, and poultry. Examples of zoonoses include tuberculosis, swine flu, rabies, HIV, and toxoplasmosis. There are several reasons for the sharp increase in zoonotic infectious diseases. In general, it can be said that the proximity of humans and animals is responsible. On one hand, humans are increasingly encroaching on the habitats of wild animals. Rainforests are being cleared to extract raw materials and create new areas for livestock farming.  When cattle graze in areas where wild animals’ habitats begin, there is increased contact between the animals, as well as between wild animals and humans. This allows pathogens to move out of their historical distribution areas. Further spread of pathogens is facilitated by factory farming, feeding animals with meat and bone meal, climate change, and the associated global warming. For example, the Anopheles mosquito has been migrating further north, giving malaria, a disease primarily spread in the tropics and subtropics, the opportunity to spread to new regions. Other factors that determine how quickly a pathogen can spread include its adaptability and reproductive rate, its evolutionary development, transmission routes, the number of hosts available for infection, and the population's susceptibility, often due to wealth or poverty.  Protecting habitats and biodiversity is a central factor in preventing the spread of new infectious diseases. When we destroy biodiversity, we can encourage the reproduction of species that are most likely to transmit new diseases to us. At the same time, there is evidence that these same species are also the best transmitters of existing diseases. Rodents and certain bats, for example, spread further when we destroy their natural habitats. And they are the ones most likely to transmit pathogens: The more we disrupt forests and natural habitats, the greater the danger we face. |
|  | 14 | **Soybean farming vs. meat consumption** In some cases, rainforests are actually cleared to create areas for growing soybeans, which are then consumed by humans. However, much less soy is needed to provide the same amount of calories for human soy nutrition as it takes via the "roundabout" route through meat.  Additionally, you should ensure that soy products come from sustainable farming in Europe. An overview can be found with the Soy Sustainability Assurance program. Unlike meat production, no antibiotics are used in the production of soy products like tofu. CO2 emissions are considerably lower. Therefore, it is helpful for the global climate if you replace the meat on your plate with tofu or other comparable plant-based proteins. |
|  | 18 | **Importance of transportation behavior** Have you ever wondered how much CO2 is emitted by cars, or whether electric vehicles are really the cleaner alternative?  In 2019, transport was responsible for about a quarter of the total CO2 emissions in the EU, with 71.7% of this coming from road transport, according to the European Environment Agency. Transport is the only sector where greenhouse gas emissions have increased over the past three decades, with a rise of 33.5% between 1990 and 2019. Reducing CO2 emissions from transport will not be easy, as the rate of reduction has slowed. Current projections estimate that transport emissions will only decrease by 22% by 2050, far behind current targets. Road transport accounts for about a fifth of the EU's emissions. CO2 emissions from passenger transport vary significantly depending on the mode of transport. Passenger cars are major polluters, responsible for 61% of total CO2 emissions from EU road transport. In 2018, the average occupancy rate was just 1.6 people per car in Europe. Increasing this through car-sharing or shifting to public transport, cycling, or walking could help reduce emissions.  For more information see: https://www.eea.europa.eu/en/analysis/publications/environmental-statement-report-2022 |
|  | 36 | **Buying seasonal fruits and vegetables** It’s worth it! When fruits and vegetables are grown in Switzerland, they are typically seasonal and come from your region. This makes them especially fresh and taste the best. Imported fruits are often not optimally ripe. They have to travel long distances and are therefore picked earlier. Fruits and vegetables from Europe almost always come by truck or ship. As a result, they cause more greenhouse gas emissions than local goods. Fruits and vegetables from other continents often come by airplane, which generates even more CO2. Thus, the following rule of thumb applies: Fruits and vegetables from Switzerland > Europe > another continent. Here’s the link: https://www.tandfonline.com/doi/abs/10.1080/15487733.2013.11908111 |
| **Quizzes** | | |
|  | 31 | **Quiz: Which mode of transportation causes the highest greenhouse gas emissions in the EU?** A. Rail transport B. Air transport C. Water transport D. Road transport  Answer: Overall, it is road transport with 71.7%  Source: https://www.eea.europa.eu/en/analysis/publications/environmental-statement-report-2022 |
|  | 6 | **How is the term "sustainable" defined?** A. The use of climate-friendly technologies B. A balance between meeting today's needs and the needs of the future C. The use of alternative energy  Answer: B is correct  Source: https://www.eea.europa.eu/en/analysis/publications/environmental-statement-report-2022 |
|  | 33 | **What actually is the greenhouse effect?** A. A phenomenon where gases allow sunlight to enter the Earth's atmosphere but make it difficult for it to leave again B. A process in which plants convert solar energy into chemical energy C. Slow warming of the Earth  Answer: A is correct  Source: https://www.un.org/en/climatechange/science/key-findings |
|  | 27 | **Fishing - which statement(s) about fishing is/are false?** A. Fishing has wiped out 90% of the large fish worldwide  B. Fishing kills 30,000 sharks per hour  C. Plastic straws make up 30% of the plastic that ends up in the oceans  D. 300,000 dolphins, whales, and porpoises are killed by fishing each year   Answer: Only C is false, they make up only 0.03%, but many straws still end up in the ocean  Source: https://www.seaspiracy.org/ |
|  | 24 | **Water consumption in meat production**  The production of the meat in a hamburger uses on average as much water as... A. a two-second shower  B. a two-minute shower  C. two showers  D. two months of daily showers  E. a year of daily showers   Answer: D is correct  Source: https://www.energy.gov/articles/quiz-test-your-climate-change-iq |
|  | 21 | **What is actually global warming?** A. General warming of the Earth  B. Process in which gases trap heat in the Earth's atmosphere  C. Current phase of climate change, which involves the long-term general increase in average temperatures of the air and oceans of the Earth   Answer: C is correct  Source: https://www.nationalgeographic.org/quiz/carylsue-test-quiz/ |
|  | 11 | **Flooding as a consequence of climate change**  Every year, floods destroy houses and livelihoods and wash away agricultural areas, leading to reduced yields and hunger. How many people are affected by flooding worldwide each year? A. 40 million  B. 250 million  C. 1 million  D. 1 billion   Answer: B is correct  Source: https://www.quarks.de/ |

*Note.* In this list of Tips, Short Readings, and Quizzes on Climate Change and Health Displayed within the GROW App, the column “Day” corresponds to the day of study participation from which on this content was unlocked and therefore displayed to the user.

### Table S7

### ***Self-report Items for Animal-Based Food Consumption***

| **Item number** | | **Survey items** | | **Answer options** | |  | |  | |  | |  | |  | |  | |  | |  | |
| --- | --- | --- | --- | --- | --- | --- | --- | --- | --- | --- | --- | --- | --- | --- | --- | --- | --- | --- | --- | --- | --- |
| 1 | | Hi! It's time again to fill out your daily diary. Do you remember the instructions for completing the diet diary?  We recommend you download the instructions, so you can have a look at them anytime you are unsure. | | show instructions | | skip instruction | |  | |  | |  | |  | |  | |  | |  | |
| 2 | | For the diet diary, we distinguish the following food categories. Please note that the categories and serving sizes are slightly simplified. Moreover, we solely focus on animal products, as these have a large impact on the carbon footprint. This means that certain categories such as fruits and vegetables are not included.  Please choose the most appropriate option when indicating the portion size consumed according to your best recollection. It is not necessary to weigh your food. If you have ever consumed more than the maximum of the indicated portions, then choose the highest indicated value.  If consuming more than the maximum of the indicated portions, please choose the highest indicated value.  - Ruminant meat (beef, lamb, deer) - Pork and any processed meat - White meat (poultry) - Fish - Seafood (Shrimps, prawns, mussels) - Eggs  - Cheese - Dairies (cow's milk, yoghurt, fresh cheese) | |  | |  | |  | |  | |  | |  | |  | |  | |  | |
| 3 | | Did you consume animal products today? | | yes | | no | |  | |  | |  | |  | |  | |  | |  | |
| 4 | | (Displayed if «yes» is flagged in survey item 3)  What kind of animal products did you consume today? | | ruminant meet | | pork and processed meat | | white meat | | fish | | seafood | | cheese | | dairies other than cheese | | eggs | |  | |
| 5 | | (Displayed if flagged in survey item 4)  RUMINANT MEAT: How many servings of ruminant meat (beef, lamb, deer) did you eat today? One serving size corresponds to the size of the palm (exclusive fingers). | | I have not consumed anything from this category. | | 0.5 serving (55 grams) | | 1 serving (110 grams) | | 1.5 servings (165 grams) | | 2.0 servings (220 grams) | | 2.75 servings (275 grams) | | 3.0 servings (330 grams) | | 3.5 servings (385 grams) | | 4.0 servings (440 grams or more) | |
| 6 | | (Displayed if flagged in survey item 4) PORK AND PROCESSED MEAT: How many servings of pork and processed meat did you eat today? One serving size corresponds to the size of the palm (exclusive fingers). | | I have not consumed anything from this category. | | 0.5 serving (55 grams) | | 1 serving (110 grams) | | 1.5 servings (165 grams) | | 2.0 servings (220 grams) | | 2.75 servings (275 grams) | | 3.0 servings (330 grams) | | 3.5 servings (385 grams) | | 4.0 servings (440 grams or more) | |
| 7 | | (Displayed if flagged in survey item 4)  WHITE MEAT: How many servings of white meat did you eat today? One serving size corresponds to the size of the palm (exclusive fingers). | | I have not consumed anything from this category. | | 0.5 serving (55 grams) | | 1 serving (110 grams) | | 1.5 servings (165 grams) | | 2.0 servings (220 grams) | | 2.75 servings (275 grams) | | 3.0 servings (330 grams) | | 3.5 servings (385 grams) | | 4.0 servings (440 grams or more) | |
| 8 | | (Displayed if flagged in survey item 4)  FISH: How many servings of fish did you eat today? One serving size of white fish is equal to the whole palm including fingers. | | I have not consumed anything from this category. | | 0.5 serving (55 grams) | | 1 serving (110 grams) | | 1.5 servings (165 grams) | | 2.0 servings (220 grams) | | 2.75 servings (275 grams) | | 3.0 servings (330 grams) | | 3.5 servings (385 grams) | | 4.0 servings (440 grams or more) | |
| 9 | | (Displayed if flagged in survey item 4)  SEAFOOD: How many servings of seafood did you eat today? Example: 9 medium shrimps weigh about 110 grams. How many servings of seafood did you eat today? | | I have not consumed anything from this category. | | 0.5 serving (55 grams) | | 1 serving (110 grams) | | 1.5 servings (165 grams) | | 2.0 servings (220 grams) | | 2.75 servings (275 grams) | | 3.0 servings (330 grams) | | 3.5 servings (385 grams) | | 4.0 servings (440 grams or more) | |
| 10 | | (Displayed if flagged in survey item 4)  CHEESE: How many servings of cheese did you eat today? One serving size is equal to the surface of your upper palm. | | I have not consumed anything from this category. | | 1 serving (45 grams) | | 2 servings (90 grams) | | 3 servings (135 grams) | | 4 servings (180 grams) | | 5 servings (225 grams) | | 6 servings (270 grams) | |  | |  | |
| 11 | (Displayed if flagged in survey item 4)  DAIRIES: How many servings of other types of dairies did you eat today? Examples: - A cup of yogurt weighs between 150 and 200 grams.  - A glass of milk weighs about 200 grams. ​​ | | I have not consumed anything from this category. | | 0.5 serving (88 grams) | | 1 serving (175 grams) | | 1.5 servings (263 grams) | | 2.0 servings (350 grams) | | 2.5 servings (438 grams) | | 3.0 servings (525 grams) or more | |  | |  | |  |
| 12 | (Displayed if flagged in survey item 4)  EGGS: How many eggs did you eat today? | | 1 | | 2 | | 3 | | 4 | | 5 | | 6 or more | |  | |  | |  | |  |
| 13 | Do you have any comments you would like to add regarding today's diary before you are reaching the end of the survey? | | (open text box) | | | |  | |  | |  | |  | |  | |  | |  | |  |

*Note.* Animal-based food consumption of participants was assessed with 13 self-report items displayed in the GROW app.

### Figure S1

### *Examples of* ***Displayed Self-report Items for Animal-Based Food Consumption in the GROW App***

*
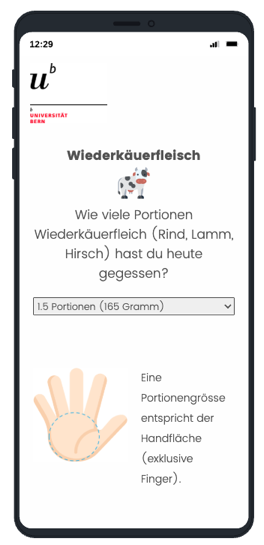

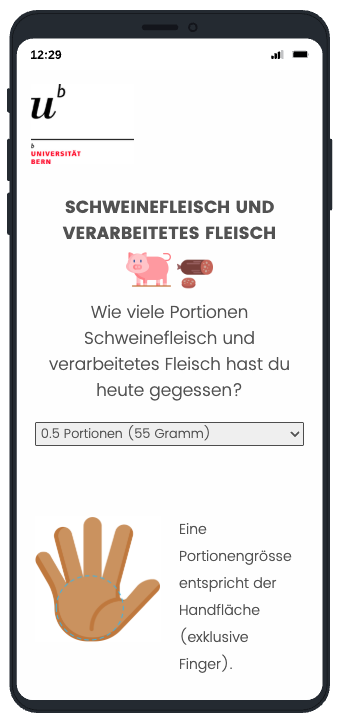


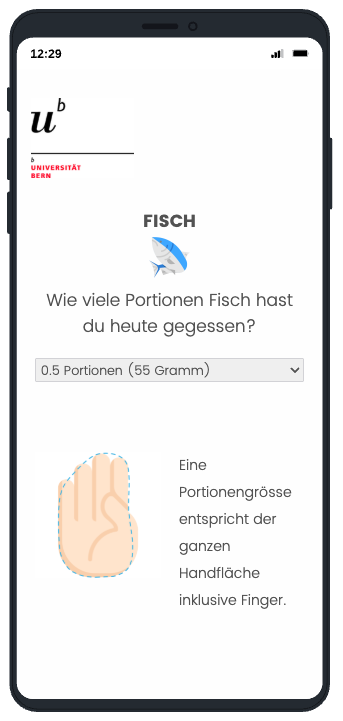

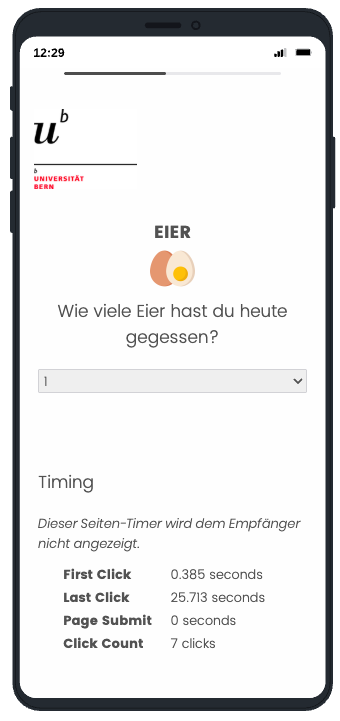
*

*Note. This figure displays examples of how the self-report survey for animal-based product consumption was displayed to participants in the GROW app.*

### Box S2

### *Calculation of Greenhouse Gas Emissions (GHGE) of Animal-Based Food Products per Serving*

To estimate diet-related greenhouse gas emissions (GHGE) per serving, we combined standard serving sizes with GHGE values reported per kilogram of food. Serving sizes (in grams per deciliter) were sourced from the Swiss Society for Nutrition based on national dietary guidelines (SGE-SSN, 2024). GHGE data (in kg CO₂-equivalents per kg food) were retrieved from the DANS SHARP-ID 2018 dataset (Mertens et al., 2019). Several food items were excluded from the GHGE-per-serving calculations due to (1) ambiguous DANS SHARP food item descriptions or (2) exceptionally high GHGE-values.

1. The DANS SHARP entries of the following meat items were too vague to be reliably categorized within our predefined food categories and were therefore excluded:

| **DANS SHARP Indicator** | **DANS SHARP-ID food item description** |
| --- | --- |
| A0EYH | Mammals and birds’ meat |
| A01QR | Meat and meat products |
| A01YG | Animal kidney |
| A04GP | Bovine and pig, minced meat |
| A023C | Ham, beef |
| A026N | Foie gras |
| A01SJ | Hare fresh meat |
| A0F1T | Animal blood |
| A0F3L | Mammals’ other organs (edible offal’s non-muscle) |
| A022R | Raw cured meat |

1. Select seafood items with exceptionally high GHGE values and low consumption frequency in the Swiss diet were removed to prevent disproportionate influence on category means:

| **DANS SHARP Indicator** | **DANS SHARP-ID food item description** |
| --- | --- |
| A02HN | Scallops, pectens |
| A02HM | Oyster, pacific cupped |
| A02HG | Oysters |
| A02FR | Lobster, European |
| A02FP | Lobsters |
| A02FV | Spiny and rock lobsters |

### Table S8

### ***Greenhouse Gas Emissions of Animal-Based Food Categories per Serving***

| *Food category* | *Number of DANS SHARP-ID food items* | *Mean GHGE [kg CO_2_-equivalent] per kg* | *Serving size in kg* | *Mean GHGE [kg CO_2_-equivalent] per serving* |
| --- | --- | --- | --- | --- |
| (1) Ruminant meat | 28 | 31.3355 | 0.110 | 3.4469 |
| (2) Pork and processed meat | 53 | 11.8279 | 0.110 | 1.3011 |
| (3) White meat | 16 | 8.5821 | 0.110 | 0.9440 |
| (4) Fish | 70 | 9.5859 | 0.110 | 1.05445 |
| (5) Seafood | 20 | 34.3577 | 0.110 | 3.7794 |
| (6) Eggs | 12 | 5.3287 | 0.110 | 0.5862 |
| (7) Cheese | 68 | 15.5760 | 0.045 | 0.7009 |
| (8) Other dairy products | 43 | 5.4798 | 0.175 | 0.9590 |

*Note.* kg = kilogram; GHGE = Greenhouse Gas Emissions. Serving sizes (in grams per deciliter) were sourced from the Swiss Society for Nutrition (SGE-SSN, 2024) based on national dietary guidelines. GHGE data (in kg CO₂-equivalents per kg food) were retrieved from the DANS SHARP-ID 2018 dataset (Mertens et al., 2019). Values were rounded to four decimal points.

### Table S9

### ***Self-report Items for Transportation Behavior***

| **Item number** | **Survey items** | **Answer options** | | | | | |
| --- | --- | --- | --- | --- | --- | --- | --- |
| 1 | Hello, it is time for you to tell us how you travelled today and what ways of transportation you used. Do you remember the instructions on how to fill out the transportation diary?  We recommend you download the instructions to look at them anytime you are unsure. | show instructions | skip instruction |  |  |  |  |
| 2 | The transport diary is designed to record your daily travels. Every day, we look at how far you have traveled by what means of transport. Please note that our list of categories of transportation modes is incomplete. Therefore, in some cases, you might be unable to report a transportation mode you have used. By traveling, we mean how you got from A to B (e.g., your way to work, your journey to your holiday destination or your route to the supermarket). We do not mean sports or other leisure activities (e.g., jogging, cycling, or walking). Please fill in the information about the distances you have travelled to the best of your recollection. |  |  |  |  |  |  |
| 3 | Did you travel today? | Yes | No, I stayed in the same place today |  |  |  |  |
| 4 | (Displayed if «yes» is flagged in survey item 3)  How did you travel today? Please select all that apply. | by bike | by motorbike | by car | by public transport (e. g., by train, tram or trolleybus) | by airplane | by foot |
| 5 | [If you do not know the number of kilometers you have travelled today, you can look it up with Google Maps.](https://www.google.ch/maps) |  |  |  |  |  |  |
| 6 | (Displayed if flagged in survey item 4)  BY FOOT: How many kilometers did you travel by foot? | Slider with a range of 6-10km | |  |  |  |  |
| 7 | (Displayed if flagged in survey item 4)  BIKE: What type of bike did you predominantly use today? | Normal Bike | E-Bike |  |  |  |  |
| 8 | (Displayed if flagged in survey item 4) BIKE: How many kilometers did you cycle? | Slider with a range of 0-40km | |  |  |  |  |
| 9 | (Displayed if flagged in survey item 4)  MOTORBIKE: Which type of motorbike did you predominantly use? | Scooter | E-Scooter | Normal Motorbike |  |  |  |
| 10 | (Displayed if flagged in survey item 4)  MOTORBIKE: How many kilometers did you ride by motorbike today? | Slider with a range of 0-200km | |  |  |  |  |
| 11 | (Displayed if flagged in survey item 4)  CAR: By what is the car you use predominantly powered? | diesel | petrol | petroleum gas, natural gas or biogas | hybrid | electricity | I don't know |
| 12 | (Displayed if flagged in survey item 4)  CAR: How many kilometers did you travel by car? | Slider with a range of 0-400km | |  |  |  |  |
| 13 | (Displayed if flagged in survey item 4)  PUBLIC TRANSPORT: What type of public transport did you use? This list of public transport is not exhaustive. If you used a mode of public transport that is not listed here, please choose 'train' instead. | train | tram | trolleybus | other |  |  |
| 14 | (Displayed if flagged in survey item 4) PUBLIC TRANSPORT: How many kilometers did you travel by public transport? | Slider with a range of 0-400km | |  |  |  |  |
| 15 | (Displayed if flagged in survey item 4)  AIRPLANE: What type of flight did you choose? | In Europe, economy class | In Europe, business class | Intercontinental, economy class | Intercontinental, business class | Intercontinental, first class | I did not fly |
| 16 | (Displayed if flagged in survey item 4)  AIRPLANE: How many kilometers did you fly by plane? | Slider with a range of 0-20’000km | | |  |  |  |
| 17 | Do you have any comments you would like to add regarding today's diary before you are reaching the end of the survey? | (open text box) |  |  |  |  |  |

***Note.*** Transportation behavior of participants was assessed with 17 self-report items displayed in the GROW app.

### Figure S2

### *Examples of* ***Displayed Self-report Items for Transportation Behavior in the GROW App***


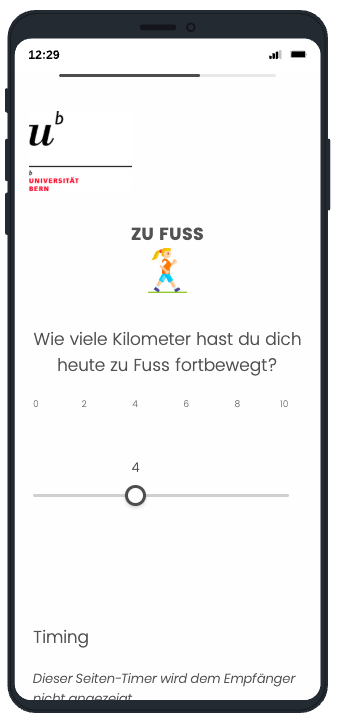

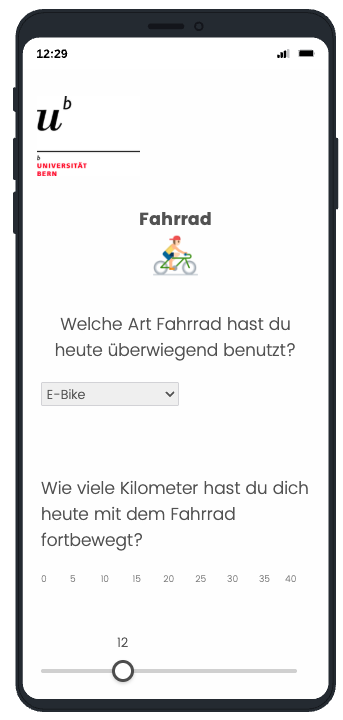


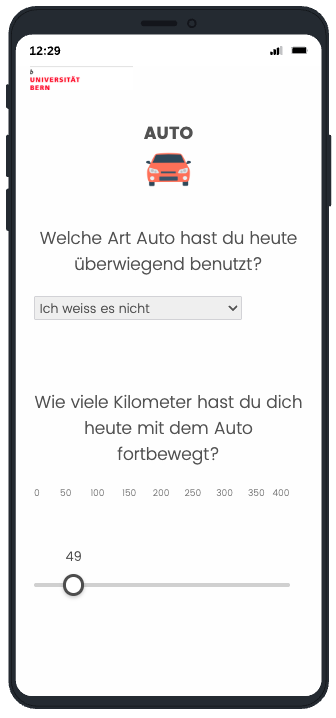

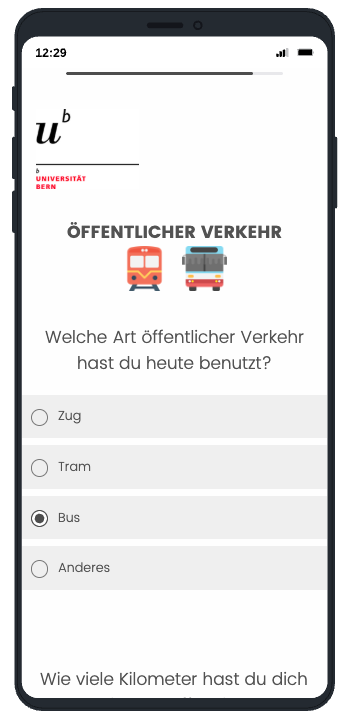


*Note.* This figure displays examples of how the self-report survey for transportation behavior was displayed to participants in the GROW app.

###

### Table S10

### ***Greenhouse Gas Emissions of Different Transportation Modes***

| **Mode of transportation** | **Vehicle type** | **Subcategory based on power source or type of flight** | **Mean GHGE [kg CO_2_-equivalent] per pkm** |
| --- | --- | --- | --- |
| By foot |  | - | 0.0000 |
| Bike | Normal bike | - | 0.0078 |
|  | E-Bike | Electricity | 0.0136 |
| Motorbike | Scooter | Petrol | 0.1037 |
|  | E-Scooter | Electricity | 0.0295 |
|  | Average motorbike | Petrol | 0.1490 |
| Car |  | Diesel | 0.1900 |
|  |  | Petrol | 0.2206 |
|  |  | Biogas / Petroleum gas / Natural gas | 0.1469 |
|  |  | Hybrid | 0.1517 |
|  |  | Electricity | 0.0822 |
|  |  | Weighted average ('don't know') | 0.2088 |
| Public transport | Weighted average |  | 0.0254 |
| Airplane |  | In Europe, Economy class | 0.2906 |
|  |  | In Europe, Business class | 0.4471 |
|  |  | Intercontinental, Economy class | 0.1902 |
|  |  | Intercontinental, Business class | 0.3930 |
|  |  | Intercontinental, First class | 0.6056 |

*Note.* kg = kilogram; pkm = person kilometer. Greenhouse gas emissions per passenger kilometer (GHGE/pkm) for each mode of transportation were sourced from the mobitool database (“mobitool-faktoren-v2.1-short-v2”, accessed July 12, 2022), managed by Switzerland’s Federal Office for the Environment. All transport modes, vehicle types, and their subcategories were directly taken from this database, with two exceptions: (1) The “Biogas / Petroleum gas / Natural gas” subcategory for cars was calculated by averaging the GHGE per pkm of the original “Biogas” and “Petroleum gas / Natural gas” categories. (2) The “Hybrid” subcategory was calculated by averaging the GHGE per pkm of the original “Plug-in Hybrid” and “Hybrid” categories. All values were rounded to four decimal places.

## Part 2. Supplementary Materials to the Results Section of the Manuscript

### ****Table S11****

### ***Social-cognitive Determinants at Baseline***

|  | **Overall**  *N* = 226 | M**otivational** **group**  *N* = 111 | M**otivational** + **Volitional group**  *N* = 115 | **Intervention group differences (p-value)*^1^*** |
| --- | --- | --- | --- | --- |
| ***Diet-related social-cognitive determinants*** | | | | |
| **Action planning** |  |  |  | 0.497 |
| Median (Q1, Q3) | 1.00 (0.33, 1.33) | 1.00 (0.33, 1.33) | 0.67 (0.33, 1.33) |  |
| Min, Max | 0.00, 2.67 | 0.00, 2.33 | 0.00, 2.67 |  |
| **Coping planning** |  |  |  | 0.881 |
| Median (Q1, Q3) | 0.33 (0.00, 1.00) | 0.33 (0.00, 1.00) | 0.33 (0.00, 1.00) |  |
| Min, Max | 0.00, 2.33 | 0.00, 2.00 | 0.00, 2.33 |  |
| **Action control** |  |  |  | 0.766 |
| Median (Q1, Q3) | 0.50 (0.00, 1.00) | 0.50 (0.00, 1.00) | 0.50 (0.00, 1.17) |  |
| Min, Max | 0.00, 3.00 | 0.00, 3.00 | 0.00, 2.50 |  |
| **Self-efficacy** |  |  |  | 0.666 |
| Median (Q1, Q3) | 1.40 (1.00, 1.60) | 1.40 (1.00, 1.70) | 1.40 (1.00, 1.60) |  |
| Min, Max | 0.00, 2.90 | 0.10, 2.90 | 0.00, 2.80 |  |
| Missing | 1 | 0 | 1 |  |
| ***Transportation-related social-cognitive determinants*** | | | | |
| **Action planning** |  |  |  | 0.851 |
| Median (Q1, Q3) | 2.00 (1.00, 2.00) | 2.00 (1.00, 2.00) | 1.67 (1.00, 2.00) |  |
| Min, Max | 0.00, 3.00 | 0.00, 3.00 | 0.00, 3.00 |  |
| Missing | 1 | 0 | 1 |  |
| **Coping planning** |  |  |  | 0.770 |
| Median (Q1, Q3) | 1.00 (0.33, 1.67) | 1.00 (0.33, 1.67) | 1.00 (0.33, 1.67) |  |
| Min, Max | 0.00, 3.00 | 0.00, 3.00 | 0.00, 3.00 |  |
| Missing | 1 | 0 | 1 |  |
| **Action control** |  |  |  | 0.315 |
| Median (Q1, Q3) | 0.83 (0.17, 1.50) | 1.00 (0.17, 1.50) | 0.67 (0.00, 1.50) |  |
| Min, Max | 0.00, 4.00 | 0.00, 4.00 | 0.00, 3.67 |  |
| Missing | 1 | 0 | 1 |  |
| **Self-efficacy** |  |  |  | 0.533 |
| Median (Q1, Q3) | 1.50 (1.10, 1.90) | 1.60 (1.10, 2.00) | 1.50 (1.10, 1.80) |  |
| Min, Max | 0.00, 3.00 | 0.00, 2.90 | 0.00, 3.00 |  |
| Missing | 2 | 1 | 1 |  |

*^1^* Wilcoxon rank sum test used for group differences in continuous variables.

### ****Table S12****

### *Weekly Participation to Intervention Modules*

|  | **Overall**  N = 226 | | **Motivational** **group**  n = 111 | | M**otivational** + **volitional group**  n = 115 | |
| --- | --- | --- | --- | --- | --- | --- |
| **Module** | **Dietary** | **Transportation** | **Dietary** | **Transportation** | **Dietary** | **Transportation** |
| Week 1 | 175 (77%) | 51 (23%) | 89 (80%) | 31 (28%) | 86 (75%) | 20 (17%) |
| Week 2 | 163 (72%) | 60 (27%) | 80 (72%) | 35 (32%) | 83 (72%) | 25 (22%) |
| Week 3 | 157 (69%) | 47 (21%) | 79 (71%) | 32 (29%) | 78 (68%) | 15 (13%) |
| Week 4 | 153 (68%) | 33 (15%) | 77 (69%) | 23 (21%) | 76 (66%) | 10 (9%) |

*Note.* Percentages are based on the sample size for each group (i.e., Overall, Motivational group, Motivational + volitional group).

### ****Table S13****

### *Sensitivity Analysis:* ***Time, Group, and Time-by-group Effects on Individual Carbon Footprint with Missing Value Imputation (Kalman filter)***

|  | **Diet-related Carbon Footprint** | **Transportation-related Carbon Footprint** | **Total Carbon Footprint** |
| --- | --- | --- | --- |
| **Fixed effects** |  |  |  |
| (Intercept) | **2.21 (0.11)** | **2.85 (0.41)** | **4.86 (0.43)** |
|  | **[2.00, 2.42]** | **[2.05, 3.65]** | **[4.01, 5.70]** |
| Time | **-0.01 (0.00)** | -0.01 (0.02) | -0.01 (0.02) |
|  | **[-0.02, -0.01]** | [-0.05, 0.02] | [-0.05, 0.02] |
| Intervention | -0.11 (0.15) | 0.34 (0.52) | 0.24 (0.55) |
|  | [-0.40, 0.18] | [-0.68, 1.36] | [-0.84, 1.33] |
| Time × Intervention | 0.00 (0.00) | -0.00 (0.02) | 0.00 (0.02) |
|  | [-0.01, 0.00] | [-0.04, 0.04] | [-0.05, 0.04] |
| Engagement with transportation module | - | 0.17 (0.31) | 0.54 (0.32) |
|  | - | [-0.44, 0.78] | [-0.08, 1.16] |
| **Random effects** |  |  |  |
| SD (Intercept) | 0.99 | 3.06 | 3.32 |
| SD (Residuals) | 1.45 | 8.19 | 8.35 |
| **Model information** |  |  |  |
| Number of observations | 7002 | 6991 | 6991 |
| R2 Marg. | 0.008 | 0.001 | 0.002 |
| R2 Cond. | 0.324 | 0.123 | 0.138 |
| ICC | 0.3 | 0.1 | 0.1 |

*Note.* Results of three multilevel models of diet-related, transportation-related and total carbon footprint. Values presented in bold are significant based on their 95% confidence intervals; *R*^2^ Marginal = the part of the variance in the outcome explained by the fixed effects; *R*^2^ Conditional = the part of the variance in the outcome explained by the full model; ICC = Intra-class correlation; Number of observations differ across models due to the exclusion of outliers.

### ****Table S14****

### *Sensitivity Analysis:* ***Time, Group, and Time-by-group Effects on Individual Carbon Footprint Including Weekend and Public Holidays as Covariate***

|  | **Diet-related Carbon Footprint** | **Transportation-related Carbon Footprint** | **Total Carbon Footprint** |
| --- | --- | --- | --- |
| **Fixed effects** |  |  |  |
| (Intercept) | **2.22 (0.11)** | **2.49 (0.41)** | **4.47 (0.43)** |
|  | **[2.00, 2.43]** | **[1.70, 3.29]** | **[3.63, 5.32]** |
| Time | **-0.01 (0.00)** | -0.02 (0.02) | -0.02 (0.02) |
|  | **[-0.02, -0.01]** | [-0.06, 0.02] | [-0.06, 0.02] |
| Intervention | -0.11 (0.15) | 0.17 (0.49) | 0.06 (0.53) |
|  | [-0.41, 0.19] | [-0.79, 1.13] | [-0.97, 1.10] |
| Time × Intervention | 0.01 (0.00) | 0.00 (0.02) | 0.00 (0.02) |
|  | [-0.01, 0.00] | [-0.05, 0.05] | [-0.05, 0.05] |
| Weekend/holiday | 0.08 (0.04) | **0.92 (0.24)** | **1.00 (0.25)** |
|  | [-0.1, 0.16] | **[0.45, 1.40]** | **[0.52, 1.49]** |
| Engagement with transportation module | - | 0.11 (0.36) | 0.53 (0.36) |
|  | - | [-0.59, 0.80] | [-0.18, 1.25] |
| **Random effects** |  |  |  |
| SD (Intercept) | 1.02 | 2.46 | 2.81 |
| SD (Residuals) | 1.56 | 8.70 | 8.86 |
| **Model information** |  |  |  |
| Number of observations | 6046 | 6035 | 6035 |
| R2 Marg. | 0.010 | 0.003 | 0.004 |
| R2 Cond. | 0.307 | 0.076 | 0.095 |
| ICC | 0.3 | 0.1 | 0.1 |

*Note.* Results of three multilevel models of diet-related, transportation-related and total carbon footprint. Values presented in bold are significant based on their 95% confidence intervals; *R*^2^ Marginal = the part of the variance in the outcome explained by the fixed effects; *R*^2^ Conditional = the part of the variance in the outcome explained by the full model; ICC = Intra-class correlation; Number of observations differ across models due to the exclusion of outliers. Public holidays included in the covariate were Ascension Day (May 9^th^, 2024), Whit Monday (May 20^th^, 2024), and the Swiss National Day (August 1^st^, 2024). Other public holidays were not included as they did not overlap with the study.

### ****Table S15****

### ***Time, Group, and Time-by-group Effects on Target Social-cognitive Determinants (No Missing Values Imputation)***

|  | **Diet** | | | | **Transportation** | | | |
| --- | --- | --- | --- | --- | --- | --- | --- | --- |
|  | **Action planning** | **Coping planning** | **Action control** | **Self-efficacy** | **Action planning** | **Coping planning** | **Action control** | **Self-efficacy** |
| **Fixed effects** |  |  |  |  |  |  |  |  |
| (Intercept) | **1.12 (0.06)** | **1.05 (0.06)** | **1.07 (0.07)** | **1.34 (0.05)** | **1.45 (0.08)** | **1.27 (0.08)** | **1.32 (0.08)** | **1.44 (0.07)** |
|  | **[1.01, 1.24]** | **[0.92, 1.17]** | **[0.94, 1.20]** | **[1.23, 1.45]** | **[1.30, 1.61]** | **[1.11, 1.42]** | **[1.15, 1.48]** | **[1.30, 1.58]** |
| Intervention | 0.10 (0.09) | 0.13 (0.09) | 0.09 (0.09) | 0.01 (0.08) | 0.05 (0.10) | 0.07 (0.10) | 0.09 (0.10) | 0.03 (0.09) |
|  | [-0.07, 0.26] | [-0.04, 0.30] | [-0.09, 0.27] | [-0.13, 0.16] | [-0.14, 0.24] | [-0.12, 0.26] | [-0.11, 0.30] | [-0.15, 0.20] |
| Time | **0.07 (0.01)** | **0.08 (0.01)** | **0.09 (0.01)** | **0.03 (0.01)** | **0.03 (0.02)** | **0.04 (0.02)** | **0.06 (0.02)** | 0.02 (0.02) |
|  | **[0.04, 0.10]** | **[0.05, 0.11]** | **[0.06, 0.12]** | **[0.01, 0.06]** | **[0.00, 0.07]** | **[0.01, 0.08]** | **[0.01, 0.10]** | [-0.01, 0.05] |
| Time × Intervention | 0.02 (0.02) | 0.00 (0.02) | 0.01 (0.02) | -0.01 (0.02) | -0.02 (0.03) | -0.01 (0.03) | -0.03 (0.03) | -0.02 (0.02) |
|  | [-0.02, 0.06] | [-0.04, 0.04] | [-0.03, 0.05] | [-0.04, 0.02] | [-0.07, 0.03] | [-0.07, 0.04] | [-0.09, 0.03] | [-0.06, 0.02] |
| Engagement with transportation module | - | - | - | - | **-0.11 (0.04)** | **-0.09 (0.04)** | **-0.16 (0.04)** | **-0.08 (0.04)** |
|  |  |  |  |  | **[-0.19, -0.03]** | **[-0.18, -0.01]** | **[-0.24, -0.07]** | **[-0.15, -0.01]** |

| **Random effects** |  |  |  |  |  |  |  |  |
| --- | --- | --- | --- | --- | --- | --- | --- | --- |
| SD (Intercept id) | 0.55 | 0.56 | 0.55 | 0.49 | 0.63 | 0.63 | 0.67 | 0.58 |
| SD (Slope – time effect)* | 0.10 | 0.09 | - | 0.08 | 0.11 | 0.12 | 0.15 | 0.09 |
| SD (Residuals) | 0.32 | 0.34 | 0.42 | 0.27 | 0.33 | 0.33 | 0.35 | 0.29 |
| **Model information** |  |  |  |  |  |  |  |  |
| Number of observations | 892 | 892 | 890 | 889 | 687 | 687 | 687 | 685 |
| R2 Marg. | 0.039 | 0.040 | 0.047 | 0.006 | 0.008 | 0.011 | 0.017 | 0.004 |
| R2 Cond. | 0.768 | 0.751 | 0.654 | 0.787 | 0.807 | 0.797 | 0.798 | 0.809 |
| ICC | 0.8 | 0.7 | 0.6 | 0.8 | 0.8 | 0.8 | 0.8 | 0.8 |

*Note.* Results of multilevel models of diet-related and transportation-related psychological determinants. Values presented in bold are significant based on their 95% confidence intervals; The standard deviation for the random slope is presented only if the random slope of time was included in the model based on BIC performance; *R*^2^ Marginal = the part of the variance in the outcome explained by the fixed effects; *R*^2^ Conditional = the part of the variance in the outcome explained by the full model; ICC = Intra-class correlation.

### ****Table S16****

### ***Time, Group, and Time-by-group Effects on Target Social-cognitive Determinants With Missing Values Imputation (Last Observation Carried Forward)***

|  | **Diet** | | | | **Transportation** | | | |
| --- | --- | --- | --- | --- | --- | --- | --- | --- |
|  | **Action planning** | **Coping planning** | **Action control** | **Self-efficacy** | **Action planning** | **Coping planning** | **Action control** | **Self-efficacy** |
| **Fixed effects** |  |  |  |  |  |  |  |  |
| (Intercept) | **1.13 (0.06)** | **1.05 (0.06)** | **1.07 (0.07)** | **1.35 (0.05)** | **1.40 (0.07)** | **1.23 (0.07)** | **1.25 (0.08)** | **1.41 (0.07)** |
|  | **[1.01, 1.24]** | **[0.93, 1.17]** | **[0.94, 1.20]** | **[1.24, 1.45]** | **[1.25, 1.55]** | **[1.09, 1.38]** | **[1.10, 1.41]** | **[1.28, 1.55]** |
| Intervention | 0.10 (0.08) | 0.13 (0.09) | 0.09 (0.09) | 0.01 (0.08) | 0.05 (0.10) | 0.06 (0.10) | 0.09 (0.11) | 0.04 (0.09) |
|  | [-0.07, 0.26] | [-0.05, 0.30] | [-0.09, 0.27] | [-0.14, 0.16] | [-0.14, 0.24] | [-0.13, 0.25] | [-0.11, 0.30] | [-0.14, 0.21] |
| Time | **0.06 (0.01)** | **0.07 (0.01)** | **0.09 (0.01)** | **0.03 (0.01)** | **0.03 (0.01)** | **0.04 (0.02)** | **0.05 (0.02)** | **0.02 (0.01)** |
|  | **[0.04, 0.09]** | **[0.05, 0.10]** | **[0.06, 0.11]** | **[0.01, 0.05]** | **[0.01, 0.06]** | **[0.01, 0.07]** | **[0.02, 0.09]** | **[0.00, 0.05]** |
| Time × Intervention | 0.02 (0.02) | 0.00 (0.02) | 0.01 (0.02) | -0.01 (0.02) | -0.03 (0.02) | -0.02 (0.02) | -0.04 (0.02) | -0.02 (0.02) |
|  | [-0.02, 0.06] | [-0.04, 0.04] | [-0.02, 0.05] | [-0.04, 0.03] | [-0.07, 0.01] | [-0.06, 0.02] | [-0.09, 0.01] | [-0.06, 0.01] |
| Engagement with transportation module | - | - | - | - | -0.06 (0.03) | -0.05 (0.03) | **-0.09 (0.03)** | **-0.05 (0.03)** |
|  |  |  |  |  | [-0.11, 0.00] | [-0.11, 0.01] | **[-0.15, -0.03]** | **[-0.10, 0.00]** |

| **Random effects** |  |  |  |  |  |  |  |  |
| --- | --- | --- | --- | --- | --- | --- | --- | --- |
| SD (Intercept id) | 0.56 | 0.58 | 0.58 | 0.51 | 0.67 | 0.67 | 0.72 | 0.61 |
| SD (Slope – time effect)* | 0.10 | 0.09 | - | 0.08 | 0.12 | 0.13 | 0.15 | 0.09 |
| SD (Residuals) | 0.30 | 0.31 | 0.40 | 0.25 | 0.26 | 0.26 | 0.29 | 0.24 |
| **Model information** |  |  |  |  |  |  |  |  |
| Number of observations | 1003 | 1003 | 997 | 994 | 984 | 984 | 984 | 982 |
| R2 Marg. | 0.033 | 0.034 | 0.040 | 0.005 | 0.003 | 0.005 | 0.008 | 0.002 |
| R2 Cond. | 0.796 | 0.788 | 0.691 | 0.811 | 0.876 | 0.870 | 0.863 | 0.871 |
| ICC | 0.8 | 0.8 | 0.7 | 0.8 | 0.9 | 0.9 | 0.9 | 0.9 |

*Note.* Results of multilevel models of diet-related and transportation-related psychological determinants. Values presented in bold are significant based on their 95% confidence intervals; The standard deviation for the random slope is presented only if the random slope of time was included in the model based on BIC performance; *R*^2^ Marginal = the part of the variance in the outcome explained by the fixed effects; *R*^2^ Conditional = the part of the variance in the outcome explained by the full model; ICC = Intra-class correlation

### ****Figure S3****

### ***Diet-related Social-cognitive Determinants Over Time***


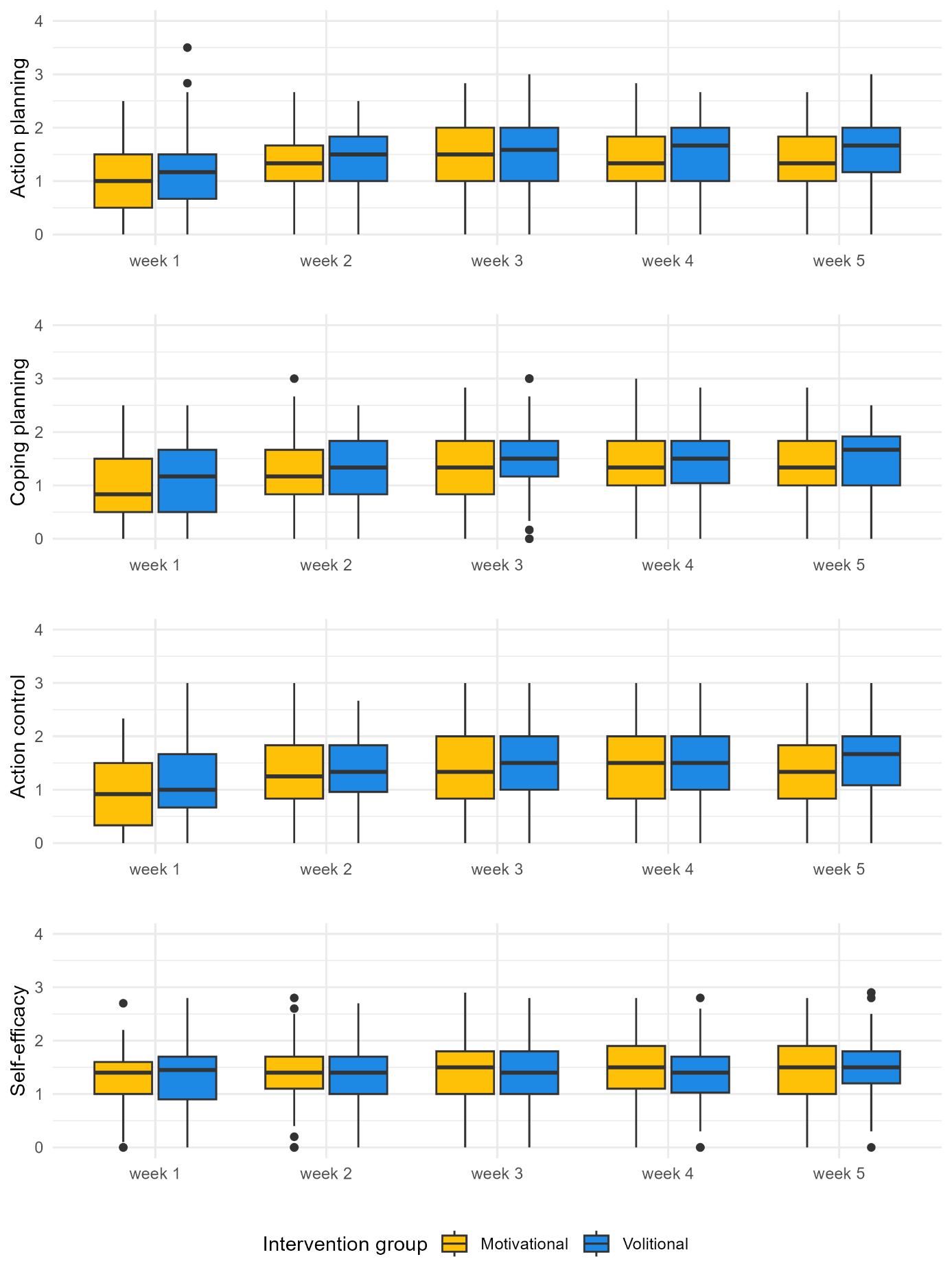


### ****Figure S4****

### ***Transportation-related Social-cognitive Determinants Over Time***


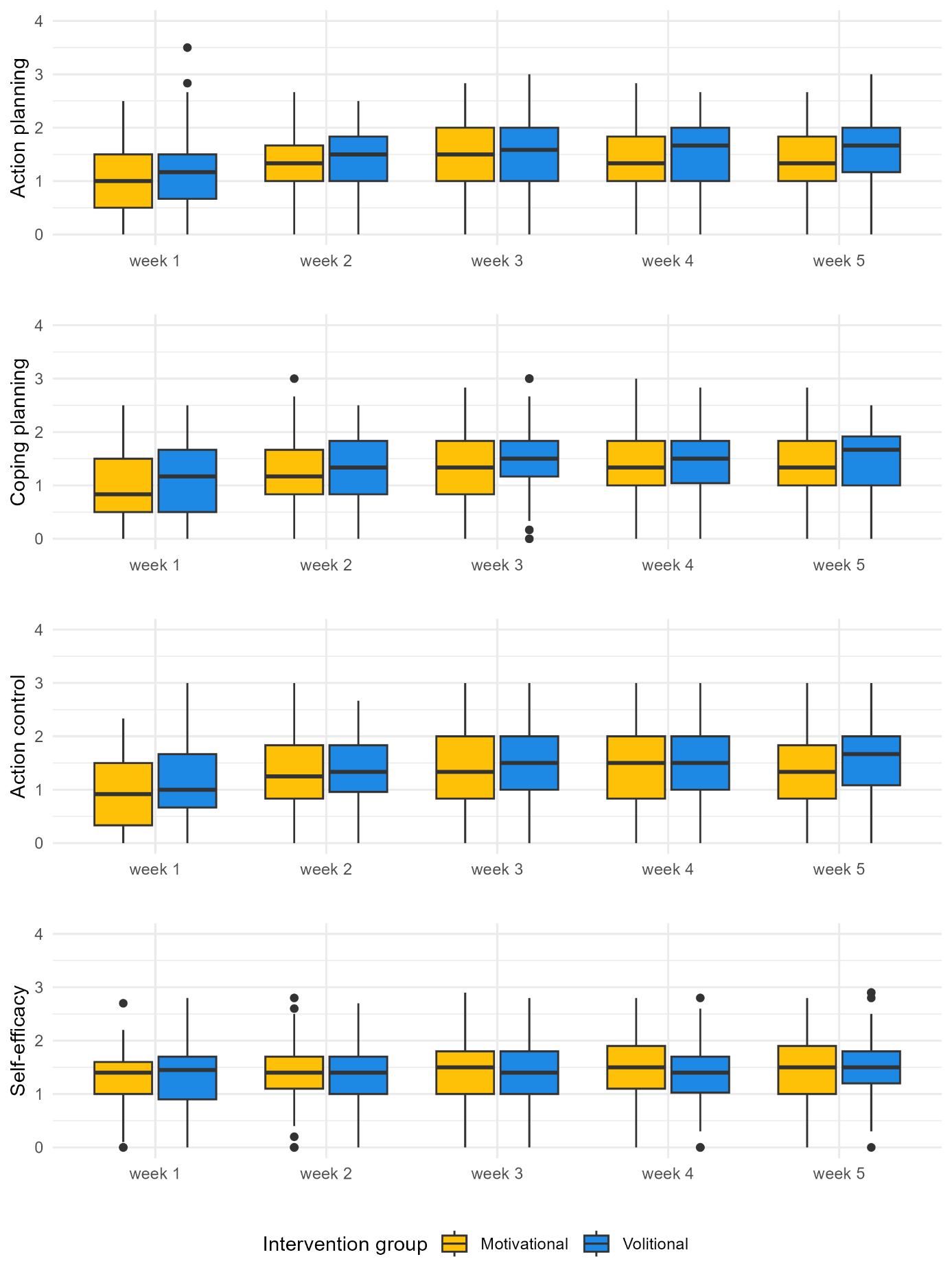


### ****Table S17****

### ***Multilevel Model of Social-cognitive Determinants on Individual Carbon Footprint***

|  | **Diet-related Carbon Footprint** | **Transportation-related Carbon Footprint** |
| --- | --- | --- |
| **Fixed effects** |  |  |
| (Intercept) | **2.00 (0.11)** | 1.53 (0.97) |
|  | **[1.77, 2.22]** | [-0.37, 3.43] |
| Time | -0.06 (0.03) | **0.74 (0.34)** |
|  | [-0.12, 0.00] | **[0.08, 1.40]** |
| Action planning (within) | -0.22 (0.14) | 0.82 (1.53) |
|  | [-0.50, 0.07] | [-2.18, 3.82] |
| Coping planning (within) | **0.27 (0.14)** | -0.96 (1.49) |
|  | **[0.00, 0.55]** | [-3.89, 1.97] |
| Action control (within) | **-0.26 (0.10)** | -1.99 (1.19) |
|  | **[-0.45, -0.07]** | [-4.34, 0.35] |
| Self-efficacy (within) | -0.21 (0.13) | -0.46 (1.41) |
|  | [-0.47, 0.05] | [-3.22, 2.30] |
| Action planning (between) | **-0.89 (0.43)** | 0.62 (2.89) |
|  | **[-1.73, -0.04]** | [-5.06, 6.29] |
| Coping planning (between) | -0.24 (0.45) | -3.99 (3.05) |
|  | [-1.13, 0.65] | [-9.98, 2.00] |
| Action control (between) | 0.63 (0.46) | 1.87 (3.10) |
|  | [-0.27, 1.54] | [-4.21, 7.96] |
| Self-efficacy (between) | -0.04 (0.26) | 0.16 (1.53) |
|  | [-0.55, 0.47] | [-2.84, 3.17] |
| **Random effects** |  |  |
| SD (Intercept) | 1.00 | 4.43 |
| SD (Residuals) | 0.76 | 7.07 |
| **Model information** |  |  |
| Number of observations | 632 | 407 |
| R2 Marg. | 0.077 | 0.030 |
| R2 Cond. | 0.665 | 0.303 |
| ICC | 0.6 | 0.3 |

*Note.* Psychological determinants of action planning, coping planning, and self-efficacy were taken from the week prior to the carbon footprint assessment. Action control was taken from the same week, as it measured action control over the previous week. Values presented in bold are significant based on their 95% confidence intervals; *R*^2^ Marginal = the part of the variance in the outcome explained by the fixed effects; *R*^2^ Conditional = the part of the variance in the outcome explained by the full model; ICC = Intra-class correlation; Number of observations differ across models due to the exclusion of outliers.

## References

Eurostat. (2025). Key figures on European transport: 2024 edition. Publications Office of the European Union. https://ec.europa.eu/eurostat/documents/15216629/20875401/KS-01-24-021-EN-N.pdf

Federal Statistical Office. (2025). Mobility and transport: Pocket statistics 2025. https://www.bfs.admin.ch/asset/en/35547668

Mertens, E., Kaptijn, G., Kuijsten, A., van Zanten, H. H. E., Geleijnse, J. M., & van 't Veer, P. (2019). SHARP indicators database (Version 2.1) [Data set]. DANS Data Station Life Sciences. https://doi.org/10.17026/DANS-XVH-X9WZ

Michie, S., Richardson, M., Johnston, M., Abraham, C., Francis, J., Hardeman, W., Eccles, M. P., Cane, J., & Wood, C. E. (2013). The behavior change technique taxonomy (v1) of 93 hierarchically clustered techniques: Building an international consensus for the reporting of behavior change interventions. Annals of Behavioral Medicine, 46(1), 81–95. <https://doi.org/10.1007/s12160-013-9486-6>

Mobitool. (2022). Mobitool factors (Version 2.1, mobitool-faktoren-v2.1-short-v2) [Data set]. https://www.mobitool.ch/

Rérat, P., & Ravalet, E. (2023). The politics of velomobility: Analysis of the vote to include cycling in the Swiss constitution. International Journal of Sustainable Transportation, 17(5), 503–514. https://doi.org/10.1080/15568318.2022.2068388

Schweizerische Gesellschaft für Ernährung. (2024). Schweizer Ernährungsempfehlungen: Langversion [Swiss dietary recommendations: Long version]. https://www.sge-ssn.ch/de/empfehlungen/offizielle-empfehlungen/ernaehrungsempfehlungen/
